# Quantifying ideological polarization on a network using generalized Euclidean distance

**DOI:** 10.1126/sciadv.abq2044

**Published:** 2023-03-01

**Authors:** Marilena Hohmann, Karel Devriendt, Michele Coscia

**Affiliations:** ^1^ Copenhagen Center for Social Data Science, University of Copenhagen, Øster Farimagsgade 5, Copenhagen, Denmark.; ^2^ Mathematical Institute, University of Oxford, Woodstock Road, Oxford, UK.; ^3^ Alan Turing Institute, Euston Road 96, London, UK.; ^4^ CS Department, IT University of Copenhagen, Rued Langgaards Vej 7, Copenhagen, Denmark.

## Abstract

An intensely debated topic is whether political polarization on social media is on the rise. We can investigate this question only if we can quantify polarization, by taking into account how extreme the opinions of the people are, how much they organize into echo chambers, and how these echo chambers organize in the network. Current polarization estimates are insensitive to at least one of these factors: They cannot conclusively clarify the opening question. Here, we propose a measure of ideological polarization that can capture the factors we listed. The measure is based on the generalized Euclidean distance, which estimates the distance between two vectors on a network, e.g., representing people’s opinion. This measure can fill the methodological gap left by the state of the art and leads to useful insights when applied to real-world debates happening on social media and to data from the U.S. Congress.

## Teaser

A previously unknown measure for estimating ideological divergence in social networks is used to study polarization.

## INTRODUCTION

Despite a multitude of studies of polarization on social media (*1*–*3*), it remains disputed whether political polarization in digital public spaces is on the rise. Many analyses conclude that polarization is rapidly advancing (*4*–*8*), while others question this interpretation (*9*, *10*). The timeliness and relevance of this issue warrant a closer look at how these claims are made and raise an important question: How can we accurately quantify the level of polarization of a social system?

The political science literature commonly distinguishes between two types of polarization: ideological and affective polarization (*11*, *12*). Ideological polarization refers to increasing ideological divergence and a reduced dialogue among individuals with differing views (*13*–*15*). Affective polarization describes in-group favoritism and out-group hostility, and it is thus concerned with the affective attitude toward others depending on their opinions (*12*, *13*, *16*). Although the two types of polarization can be mutually reinforcing (*17*–*19*), ideological polarization and affective polarization are distinct concepts, both in terms of theory and empirical measurement (*11*, *12*). While the measure of ideological polarization relies on data about the opinions of people, affective polarization also requires information about the valence of their relationships (*20*–*22*).

Here, we focus on ideological polarization in social networks; hereafter, whenever we do not qualify the term “polarization,” we refer to ideological polarization. As outlined above, ideological polarization refers to increasing ideological divergence on the one hand and increasing reluctance to engage with diverging views on the other hand (*13*–*15*, *23*). From this conceptual understanding, we derive two components of ideological polarization in social networks and an interplay between the two: A social network is more polarized than another if the opinions of its members diverge strongly (opinion component), if people with similar opinions cluster with each other in communities (structural component), and if these communities tend to organize themselves in an ideological spectrum rather than engaging with all other communities (mesoscale interplay of opinion and structure).

Since current network-based measures can only partially capture these components, we propose a measure of ideological polarization. Our measure is based on a generalized Euclidean (GE) distance measure (*24*), and it estimates how much effort it would take to travel from one opinion to another in the network.

The literature has advanced numerous ways to estimate ideological polarization, which we briefly review here. Some methods consider exclusively opinions (*25*) or reduce the complexity of the structure (*26*), which we think does not allow to properly capture what we understand as polarization. Other approaches rely on local network measures to evaluate the structure of interactions in a network (*27*–*29*). The assortativity coefficient, for instance, quantifies to what extent individuals are directly linked to like-minded others (*30*–*32*). Similarly, there are methods that assess the average opinion of the direct neighbors of an individual (*33*, *34*). However, local measures are myopic to the overall structure and would return the same estimations even if opinions are distributed in radically different mesoscale structures such as communities (*35*).

Alternative methods explicitly divide the network into two communities to determine how well they are separated from each other (*36*–*41*). While these measures can account for the network structure, a two-community partition implies an expectation of polarization that might not exist. Methods that avoid the partition phase (*42*) provide node-dependent estimates, and it is unclear how to summarize them for the whole network.

Another approach builds on the opinion formation model proposed by Friedkin and Johnsen (*43*). This measure assumes that each individual has both an internal opinion and an expressed opinion that is determined by their own internal opinion and the surrounding opinions in the social network (*44*, *45*). However, it is questionable whether individuals have stable internal opinions on a political issue since public opinion research suggests that individual-level issue opinions are often inconsistent and volatile (*46*–*48*). Apart from these conceptual considerations, this approach entails a practical problem: Social media data can only capture people’s expressed opinions but not their internal opinions on an issue (*49*). Our approach sidesteps this issue by not requiring to know the internal opinion of an individual.

Last, one could use graph neural networks (*50*), but these techniques normally provide a simple classification of whether a structure is polarized rather than quantifying the polarization level. Our approach overcomes the aforementioned issues using data available on social media: the people’s expressed opinions and their social relationships. The former is determined on the basis of social media users’ sharing behavior (*34*, *51*), and the latter is determined by downloading their connections such as, e.g., follower relationships on Twitter. We estimate the GE distance (*24*, *52*) between two opposing opinions across all the edges of the network. By doing so, we avoid using a local approach, and we do not assume a community structure by default.

In Results, we demonstrate how our approach is the only alternative we found that is sensitive to the two components of ideological polarization outlined above, as well as their interplay. Moreover, we show that our measure allows us to make useful inferences about real-world polarized systems such as political debates on Twitter or voting patterns in the U.S. House of Representatives.

## METHODS

### Definition

**Fig. 1.**
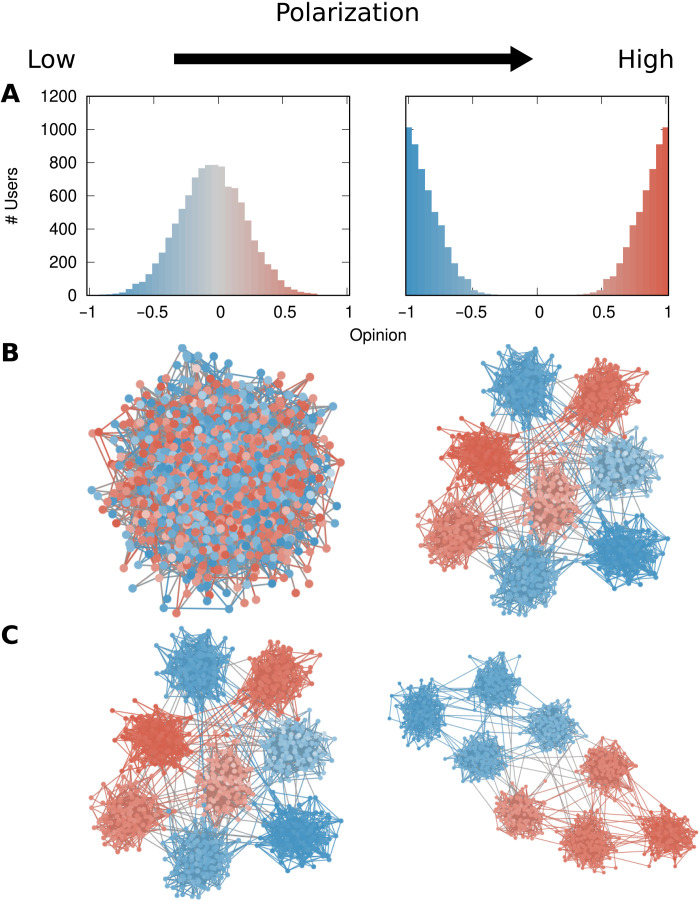
The two components of polarization in a network and their interplay. (**A**) Opinion component: Plots show the number of people (*y* axis) with a given opinion value (*x* axis and bar color). (**B**) Structural component: People are nodes, connected if they are interacting with each other. Node color represents the opinion (<0, blue; >0, red), and edge color represents the average opinion of the two connected nodes. One community is on the left and eight communities are on the right. (**C**) Opinion-structural interplay: Same legend as in (B). All communities equally interconnected on the left, each community only connected to its most similar opinion community on the right.

On the basis of the existing literature on ideological polarization, we define two components of polarization and their interplay (Fig. 1):

1) Opinion component (Fig. 1A): Traditional political science studies of ideological polarization consider whether and how people’s ideological leanings diverge (*11*, *14*, *15*): If opinions cluster in the moderate center, then polarization is low (example on the left). If they instead disperse toward the extremes, then polarization is high (example on the right).

2) Structural component (Fig. 1B): More recent approaches have emphasized the role of social connections, and especially homophily, i.e., the connections between like-minded individuals (*23*, *53*–*56*). If there is no community structure, then there is no opinion homophily, and each individual is connected and therefore exposed to many different views. In this case, polarization is low (example on the left). However, if there are clearly separated communities, then individuals are only exposed to the opinions within their community, but they are not directly exposed to other opinions, and polarization is therefore high (example on the right).

3) Mesolevel organization of the opinion-structural interplay (Fig. 1C): Opinion and network structure have largely been viewed as separate indicators of polarization. To integrate the two strands of the literature into a unified definition, we propose to also consider the interplay between the two components. We understand this interplay as follows: The same opinions and the same communities can give rise to different levels of polarization depending on the mesolevel organization of the system. Communities that can freely interlink regardless of their opinions indicate a lower level of polarization (example on the left) than if communities organize in progressively more extreme echo chambers (example on the right).

Since ideological polarization has previously been described both in terms of opinions and network structure, we consider these two components to be distinct yet related aspects of polarization. This view is supported by some of the real-world examples that we examine as we can show that opinion and structure are correlated in the Twitter networks that we analyze (see section S8). We therefore argue that it is important for a measure of polarization to consistently capture the two components and their interplay in a single measure.

### Formulation

We refer to our polarization measure as δ_*G*,*o*_. The measure requires two inputs: the graph structure *G* and a vector of opinions *o*. δ_*G*,*o*_ takes values from 0, which implies no polarization at all, to an arbitrary positive number. The higher the value, the more polarized the network is.

The first parameter is a simple graph *G* = (*V*, *E*), with *V* being the set of nodes and *E* ⊆ *V* × *V* as the set of connections, i.e., node pairs (*i*, *j*) with *i*, *j* ∈ *V*. For simplicity, we assume edges in *E* to be unweighted and undirected [(*i*, *j*) = (*j*, *i*)], but our approach can consider edge weights. There are a few mandatory requirements on *G*. *G* must not contain self-loops, edges connecting a node with itself. It must also be connected, i.e., there must be at least one path between any two nodes in the graph. The polarization δ_*G*,*o*_ cannot be estimated if these conditions are not satisfied.

The second parameter is the vector of opinions *o*. This vector *o* must have length ∣*V*∣, i.e., record a single value per node. We impose a convention on *o*: The opinion values must be bounded between −1 (the most extreme opinion on one side) and +1 (the most extreme opinion on the other side). In such a vector, 0 represents perfect neutrality between the two opinions. In real-world U.S. politics data, −1 could be an extreme Democrat, and +1 could be an extreme Republican, with 0 as perfect independents.

δ_*G*,*o*_ is based on a solution (*24*) to the node vector distance problem (*57*). In GE, one can use the pseudo-inverse Laplacian to estimate the effective resistance (*58*) between two arbitrary vectors of length ∣*V*∣ recording a variable per node of the network. We recall that the Laplacian matrix is *L* = *D* − *A*, with *A* being the adjacency matrix of *G* and *D* being the diagonal matrix containing the degrees of the nodes of *G*. Thus

$$L_{ij}=\{\begin{array}{cc} d_{i} & \mathrm{if}\;i=j\\ -1 & \mathrm{if}\;(i,j)\in E\\ 0 & \mathrm{otherwise} \end{array}$$

To estimate the effective resistance, we need to invert *L*, but *L* is singular and therefore cannot be inverted. For this reason, we take the Moore-Penrose pseudo-inverse of *L*, symbolized as *L*^†^. Then, for two arbitrary node vectors *a* and *b*

$$\delta_{G,a,b}=\sqrt{\left(a-b{)}^{T}L^{†}(a-b\right)}$$

Previous work shows that this formula gives a good notion of distance between vectors *a* and *b* on a network (*24*, *57*). Specifically, it can recover the infection and healing parameters in a susceptible-infected-susceptible or susceptible-infected-recovered (SIR) model by comparing two temporal snapshots of an epidemic—a more infectious disease with faster recovery covers more space across a social network in the same amount of time.

**Fig. 2.**
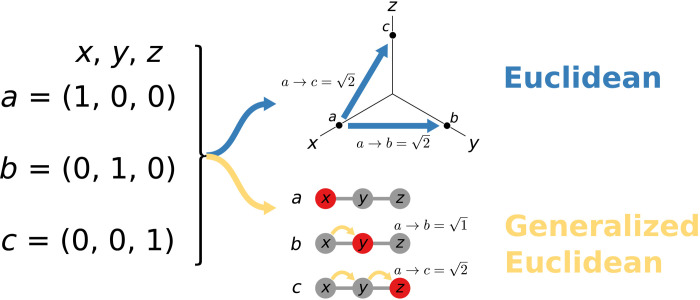
Difference between generalized and plain Euclidean. We start from three vectors *a*, *b*, and *c* in 3D spaces *x*, *y*, and *z*. The blue arrow points at a Euclidean space, with labeled dots showing the positions of vectors *a*, *b*, and *c* in independent dimensions *x*, *y*, and *z*. The yellow arrow points to an *x*, *y*, or *z* space defined on graph *G*, and the node color represents the values for vectors *a*, *b*, and *c* (red equals to 1 and gray equals to zero).

Figure 2 shows this intuition in the simplest possible scenario. We have three 3D (three-dimensional) vectors *a* = (1,0,0), *b* = (0,1,0), and *c* = (0,0,1). We use *x*, *y*, and *z* to refer to the three spatial dimensions. In the traditional Euclidean case, following the blue arrow, the three spatial dimensions are uncorrelated, and thus, moving an equal amount in each direction contributes equally to the distance measures. Thus, *a* is equidistant from *b* and *c*, at a distance of $\sqrt{2}$.

However, we can use a graph *G* to express relationships between the dimensions as we do if we follow the yellow arrow in the figure. In that case, intuition tells us that *b* is closer to *a* than *c*, as the nodes with a value of 1 are two steps away in *c* and only one step away in *b*; $\delta_{G,a,b}=\sqrt{1}$ and $\delta_{G,a,c}=\sqrt{2}$.

To use GE for the purpose of estimating polarization, we need to split the vector *o* in two vectors: *o*^+^ and *o*^−^. *o*^+^ contains all positive opinions and zero otherwise; *o*^−^ contains the absolute value of all negative opinions and zero otherwise. Once we do that, our δ_*G*,*o*_ measure of polarization becomes

$$\delta_{G,o}=\sqrt{{\left(o^{+}-o^{-}\right)}^{T}L^{†}(o^{+}-o^{-})}$$

The unit of our measure is the step or, to be more precise, its square root. This is the same unit as the one used by, e.g., the shortest paths: If one needs to cross five edges to go from node *i* to node *j*, then *i* and *j* are five steps away from each other. In practice, one can interpret δ_*G*,*o*_ as the average “distance” between randomly sampled nodes in *o*^+^ and *o*^−^, weighted by how strongly these nodes hold their opinion (e.g., the distance between two nodes with opinions +1 and −1 is weighted higher than if the nodes had opinions −0.1 and +0.1). The units of this expected distance are “steps,” and, as further discussed in Analytical Approach and section S4, the notion of distance in this interpretation is the so-called effective resistance.

We can see how δ_*G*,*o*_ considers all the factors that we outlined in the previous section. The more well separated the communities are and the more they are organized at the mesolevel in an opinion spectrum, the more steps are necessary to traverse the network. The larger the opinion difference *o*^+^ − *o*^−^, the more these steps are weighted.

## RESULTS

We compare δ_*G*,*o*_ only with measures accepting the same input and providing the same output. Hence, methods working with signed networks (*20*–*22*, *59*) or with expressed and internal opinions (*44*, *45*, *49*) or providing a simpler classification output in the form of a yes/no value (*50*) are beyond the scope of this paper.

We specifically look at opinion assortativity (ρ_*G*,*o*_) (*32*), random walk controversy (RWC*_G_*) (*37*), density plots of opinion against average neighbor opinion (*33*), and boxplots of opinion against average opinion of the set of influenced nodes in an SIR model (*34*). Section S1 includes details of how these measures are calculated.

### Synthetic data

We now show how δ_*G*,*o*_ is sensitive to the components of our definition of polarization, while the alternative ways of estimating polarization are insensitive to at least one of those factors. We follow the rows in Fig. 1 one by one and show how the δ_*G*,*o*_ values and the alternative measures evolve over those dimensions. All numeric values reported in the figures that follow are the averages of 25 independent runs. All pairs of δ_*G*,*o*_ scores presented in the main text are statistically different, with the minimum *z* score of the difference between any of the values shown being 3.4 corresponding to a one-tailed *P* < 0.001. The density and box plots are taken from one representative run. The details on how we generate the various *G* and *o* values, as well as the relevant parameters, are provided in Analytical Approach. Sections S2 and S3 contain additional tests on the behavior of δ_*G*,*o*_ including its values for some interesting edge cases.

#### The opinion component

**Fig. 3.**
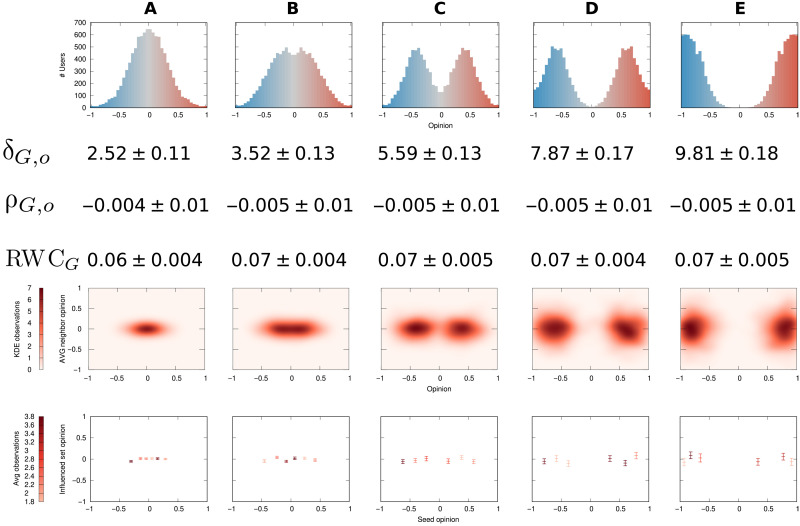
The opinion component of polarization. Each row (top to bottom): Distribution of *o* values, the number of nodes (*y* axis) with a given opinion (*x* axis and bar color); values of δ_*G*,*o*_, ρ_*G*,*o*_, and RWC*_G_* with their SDs across 25 independent runs; kernel density estimation (KDE) maps of opinions (*x* axis) and average neighbor opinion (*y* axis); boxplots of seed opinion (*x* axis) and average opinion of the influenced set after an SIR propagation (*y* axis). The boxplots show the average for the middle tick and ±SE for the top/bottom ticks. In the bottom two rows, color (from bright to dark) is used proportionally to the number of observations within the data point. Changes in the distribution of opinions lead to progressively increasing polarization through columns (**A**) to (**E**). The graph *G* (not depicted) has no communities.

In Fig. 3, we start with a network without a community structure, in which the opinions distribute normally in the opinion spectrum (leftmost plot) and randomly over the network. This is a state of low polarization. As we move from Fig. 3A to Fig. 3E, we create more and more polarization in the opinion vector *o*, keeping *G* as a random graph without communities. The second row shows that the δ_*G*,*o*_ values grow by a factor of almost 4, implying a substantial increase in polarization. This corresponds to our intuition about the opinion component of polarization.

Neither assortativity (ρ_*G*,*o*_, third row) nor RWC*_G_* (fourth row) is able to capture this change. All their values are not statistically different from each other. This is because a random graph has zero expected assortativity (see section S1), while RWC*_G_* must bisect the network into two communities, regardless of how extreme the opinion difference is. The density maps of the average neighbor opinion (fifth row) and the average influenced set opinion (sixth row) are able to capture the differences in the opinion value distributions.

#### The structural component

**Fig. 4.**
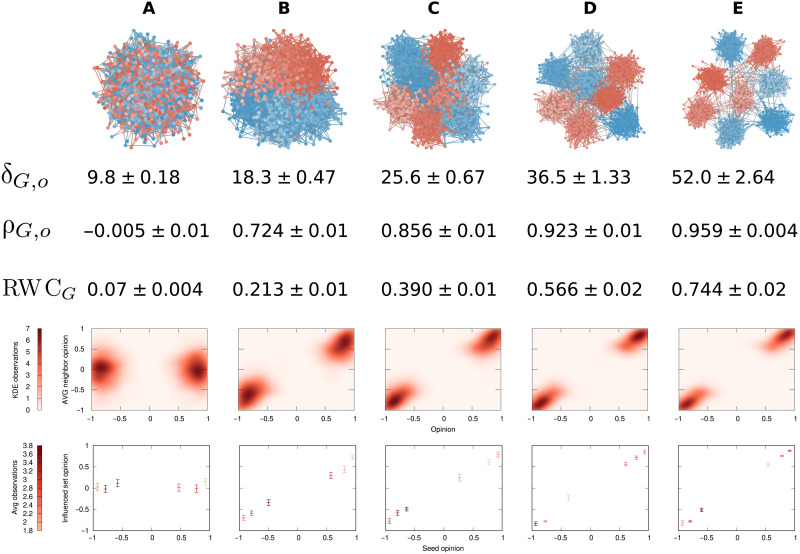
The structural component of polarization. Same legend as that of Fig. 3. Structural changes lead to progressively increasing polarization through columns (**A**) to (**E**). *p*_out_ values: (A) 0.0085, (B) 0.0024, (C) 0.0012, (D) 0.0006, and (E) 0.0003.

Next, in Fig. 4, we take the most polarized opinion vector *o* from Fig. 3—the distribution in the top row of Fig. 3E—and we investigate the structural component of polarization. We create eight communities in the network, each of which has a high degree of opinion homophily. As we move from Fig. 4A to Fig. 4E, we change the connection probabilities of the nodes inside the network. We decrease *p*_out_, the probability that a node will connect to a node in a different community. We then increase *p*_in_, the probability of a node connecting to a node in the same community so that all networks in Fig. 4 have the same expected number of edges.

The larger the difference between *p*_out_ and *p*_in_, the higher the polarization, driven by the assortative communities in the structural component. We see that the values of δ_*G*,*o*_ (second row) follow our expectation, growing by a factor of around 5. Thus, we can conclude that the measure is also sensitive to the structural component of polarization, not only to the opinion component.

Assortativity (ρ_*G*,*o*_, third row) is able to distinguish between the five networks, but it is overly sensitive to relatively small initial changes to the random structure, downplaying the subsequent emergence of strong communities. The difference between Fig. 4A and Fig. 4B is more than three times as large as the difference between Fig. 4B and Fig. 4E. This shows that, while assortativity can catch structural separation, it makes it difficult to distinguish weak communities from strong ones. The same can be said for the density maps of the average neighbor opinion (fifth row) and the average influenced set opinion (sixth row). RWC*_G_* (fourth row) picks up structural separation well.

#### The opinion-structural interplay

**Fig. 5.**
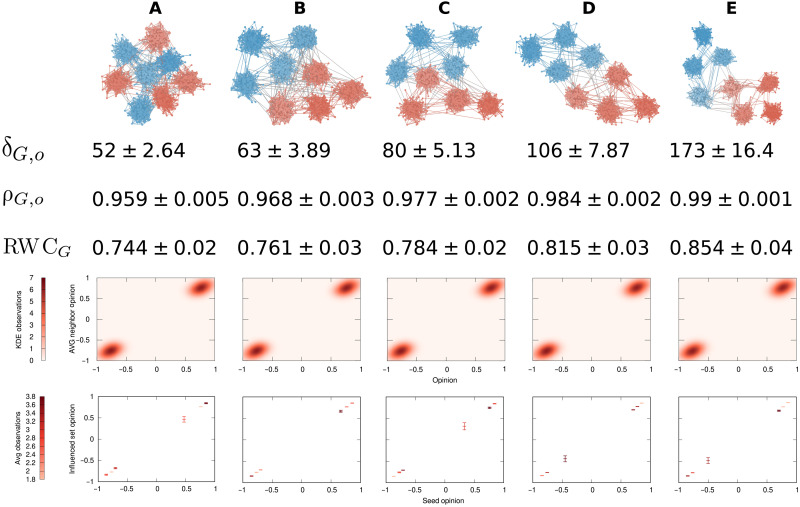
The opinion-structural interplay. Same legend as Fig. 3. Changes in the opinion-structural interplay lead to progressively increasing polarization through columns (**A**) to (**E**). Each community in the network is connected to its (A) seven, (B) five, (C) four, (D) three, and (E) two closest communities in terms of average opinion.

Last, in Fig. 5, we observe what happens when we modify the opinion-structural interplay at the mesolevel. To do so, we set some *p*_out_ values to zero. Specifically, each community gets progressively more and more isolated from the rest of the network as they preferentially disconnect from communities with a larger opinion difference. This mesoscale structure is something that we observe empirically, as we show later when looking at data from actual debates on Twitter.

The network in Fig. 5A is roughly equivalent to the one in Fig. 4E, where all communities connect to each other, and where the opinion distribution has values clustered around −1 and +1. Starting from Fig. 5B to Fig. 5E, we lower the number of connected neighboring communities from five to two.

Again, moving from Fig. 5A to Fig. 5E implies increasing levels of polarization, as it gets progressively harder for people to be exposed to differing points of view. This is reflected by a threefold increase in the value of δ_*G*,*o*_. The large difference for each column shows that the measure is sensitive to the structural changes at the mesolevel.

Assortativity (ρ_*G*,*o*_, third row) and RWC*_G_* (fourth row) are not particularly sensitive to the mesolevel organization of the network, certainly not as much as they are to the structural component alone. Assortativity only changes at the second significant digit and always scores values near the maximum of +1. On the other hand, RWC*_G_* is prone to misclassification, as the SDs show that there is an overlap between the higher bound of one level (for instance, Fig. 5A) and the lower bound of the following one (in this case, Fig. 5B). However, both measures do a reasonable job at capturing the mesolevel organization of the opinion-structural interplay.

The density maps of the average neighbor opinion are indistinguishable from each other (fifth row). This is because they exclusively look at local information, and they are blind to the mesolevel organization of the network. The average influenced set opinion (sixth row) could, in principle, capture the mesolevel organization as it is not bound by looking at direct neighbors but allows the influenced set to percolate through the structure. However, the communities are too large and too well separated for this to happen in practice, and the differences between each plot from Fig. 5A to Fig. 5E are minimal.

From Figs. 3 to 5, we can conclude that δ_*G*,*o*_ is the only measure sensitive enough to recognize each further example as a part of a continuum of increasing levels of polarization. δ_*G*,*o*_ captures the opinion and structural components, as well as their interplay happening at the mesolevel of the network. We support this statement by showing, in section S3, how δ_*G*,*o*_ varies smoothly across all the parameter values used to generate our synthetic data.

The alternative measures lack sensitivity to at least one aspect of polarization. Assortativity and RWC*_G_* are blind to the opinion component and overemphasize the structural component over the opinion-structural interplay, while density maps of the average neighbor opinion and the average influenced set opinion in an SIR propagation do not capture the opinion-structural interplay at the mesolevel and overemphasize the opinion component over the structural one.

### Applications

We now turn to looking at real-world networks to show the insights one could gather from using δ_*G*,*o*_. We compare different political debates happening on Twitter including the 2020 U.S. presidential election and the evolution of U.S. representatives over time.

#### Twitter debates

**Fig. 6.**
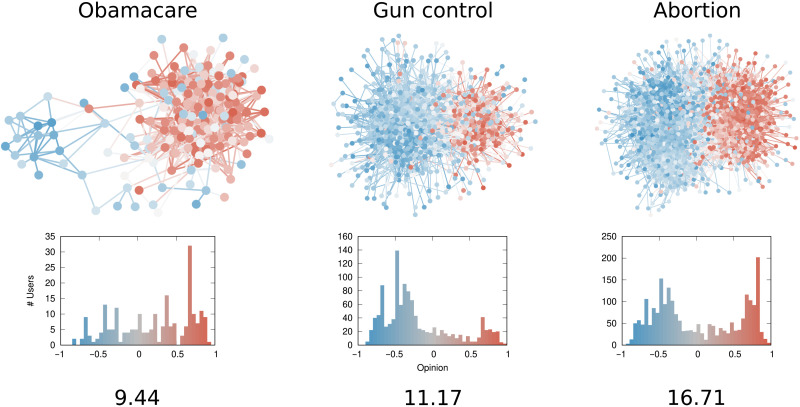
The Twitter debate networks. From top to bottom row: Network topology, users as nodes, interactions as edges, and opinions as colors of both nodes and edges; opinion distribution, the number of users (*y* axis) with a given opinion (*x* axis and bar color); δ_*G*,*o*_ score.

Figure 6 shows examples of three debates happening on Twitter in the mid-2010s. The node color reflects ideological leaning on a United States–focused liberal (blue) to conservative (red) scale. These center on three topics in the U.S. political context, which had been discussed by Twitter users between 2015 and 2016: the U.S. Medicare reform known as Obamacare, gun control, and abortion.

δ_*G*,*o*_ shows moderate levels of polarization with values between 9 and 17. The least polarized debate is about Obamacare, while the abortion debate is the most polarized. We call these levels of polarization “moderate” for several reasons. First, most opinions in the Obamacare network are uniformly distributed over the entire spectrum, leaving structure as the main source of polarization. The gun control network has more diverging and extreme opinions, but the distribution of ideological leanings is heavily skewed to the left, reducing polarization; polarization is low if most people agree on a position, even if it is a relatively extreme one. In this case, the vast majority of users are located left of center, and as a consequence, the δ_*G*,*o*_ score is reduced. The abortion debate is the most polarized because it has both high opinion divergence and roughly equally sized clusters. The score is still moderate because there is a high number of connections between the clusters, showing a level of communication between the faction that reduces overall polarization—3% of all the edges of the network are between a “red” and a “blue” node, while this figure is below 2% for both the Obamacare and gun control debates.

Note that the abortion network actually has a mesoscale organization with subcommunities inside the main two opposing communities, as we detect via a stochastic blockmodel (SBM) community discovery in section S8. This provides support to our definition of polarization that includes an opinion-structural mesoscale interplay.

Note that δ_*G*,*o*_ is scale invariant as we show in section S2. It follows that differences in the polarization scores cannot be ascribed simply to the size of the network in terms of the number of nodes.

#### Twitter elections

**Fig. 7.**
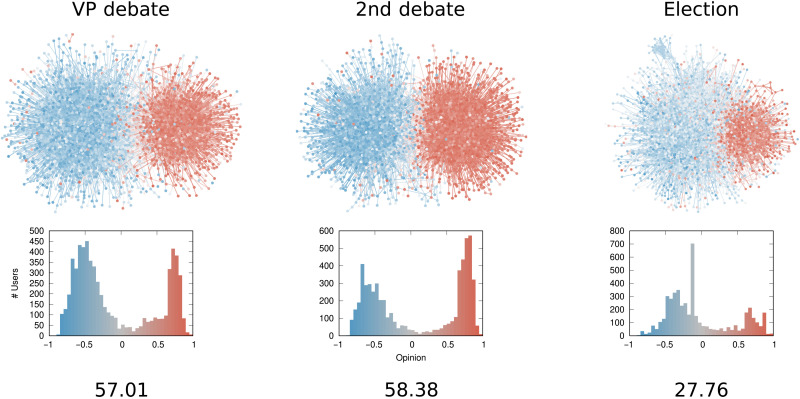
The Twitter election networks. From top to bottom row: Network topology, users as nodes, interactions as edges, and opinions as colors of both nodes and edges; distribution of ideological leanings, the number of users (*y* axis) with a given opinion (*x* axis and bar color); δ_*G*,*o*_ score.

Figure 7 shows the progression of the U.S. presidential election in 2020. The networks center on the vice presidential debate (7 October 2020), the second presidential debate (22 October 2020), and the election day (3 November 2020).

δ_*G*,*o*_ shows high levels of polarization for the second debate and the vice presidential debate. Both networks contain two extremely separated clusters with fewer than 1% of edges between them. Moreover, the opinion values are distributed toward the extremes. This explains why the scores are higher than the ones we show in Fig. 6. During the 2020 election, users held opinions farther from each other and stopped interacting with disagreeing users.

Election day has substantially lower polarization due to a noticeable spike in the neutral portion of the opinion spectrum. This is caused by the necessity of sharing raw election result updates, which come from neutral and factual sources. The most shared domain during that period is from Associated Press, which has a moderate opinion value of −0.13 and is responsible for the noticeable peak in the opinion distribution. This suggests caution when estimating polarization scores in a context where people are both discussing opinions and hard facts at the same time.

#### U.S. house of representatives

We build the networks using voting records from the U.S. House of Representatives (*60*). We connect two congressmen if they cast the same vote on the same bill for a substantial number of times; the Analytical Approach provides more details. The *o* vector is their DW-NOMINATE score (*61*), an established way of quantifying their political leaning. We do not consider data from the Senate because senators cannot co-vote with members of the House: Including them would create a disconnected network. We observe comparatively low δ_*G*,*o*_ values for two reasons. First, although *G* has two opposing dense communities, the network has a small diameter and average path length of approximately 1.5 to 1.95 (see table S4). This means that extreme congressmen in either community are separated by less than two steps on average, leading to low structural separation. Second, depending on the congress, 67 to 93% of the DW-NOMINATE scores are between −0.5 and 0.5, which suggests low opinion divergence as well, because the opinion values predominantly cover a smaller portion of the available [−1, + 1] interval.

**Fig. 8.**
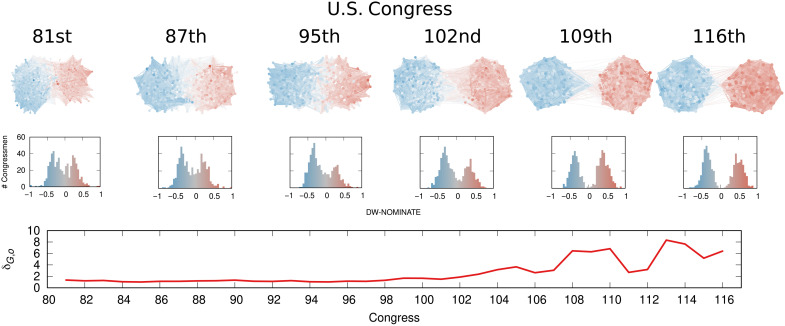
Polarization in the U.S. House of Representatives. From top to bottom row: Network topology, congressmen as nodes, co-voting relationships as edges, and opinions as colors of both nodes and edges; opinion distribution, the number of congressmen (*y* axis) with a given opinion (*x* axis and bar color); δ_*G*, *o*_ score. For the network and opinion rows, we show 6 examples of the 36 networks analyzed.

Notwithstanding these characteristics, the U.S. Congress has been viewed as an example of polarization escalation (*62*). Figure 8 supports this view. Up until the 98th Congress (1983–1985), polarization was almost nonexistent, with δ_*G*,*o*_ scores around 1. Ever since the 98th Congress, δ_*G*,*o*_ scores have been on the rise to a maximum of more than 8.

This can be considered a high score, given the caveats we presented about how *G* and *o* are built. We should not compare these scores directly with the ones obtained from Twitter since the way of estimating *o* is vastly different. To contextualize the score, we can pick extreme Democrats and Republicans in the 116th Congress (2019–2021) and calculate the score that we would get if they represented the entirety of their parties. If we perform this experiment using James McGovern for Democrats and Matt Gaetz for Republicans, then we get a score of 14. This can be considered close to the maximum, as McGovern is part of the most left-leaning caucus of the House (the Congressional Progressive Caucus) and Gaetz is part of the most right-leaning one (the Freedom Caucus). They both have extreme DW-NOMINATE scores as well. On the other hand, the most moderate possible pair in the 116th Congress according to DW-NOMINATE is Ben McAdams and Brian Fitzpatrick who were members of the centrist caucuses Blue Dog and Main Street Partnership. If they were composing the entirety of their parties, the polarization score would be a mere 0.2.

According to our measure, the most polarized House in the post–World War II history was the 113th (2013–2015), which coincided with the beginning of Barack Obama’s second term, plus a debt-ceiling crisis following the full application of the Affordable Care Act (Obamacare), the 2014 Russo-Ukrainian conflict, strong debates about immigration reforms, and a controversial escalation of U.S. military action in Syria and Iraq against Islamic State of Iraq and Syria (ISIS), among other things.

## DISCUSSION

Here, we tackled the issue of estimating the level of polarization in a social network. We ask how polarized a system is given the set of social connections and the opinions of all the individuals in the network. We decompose the polarization question in two main components and an interplay factor: how varied the opinions are (opinion component), how assortative the communities are (structural component), and how communities organize at the mesolevel of the network (opinion-structural interplay).

Intuitively, our estimate is based on the network distance between all pairs of disagreeing individuals, weighted by how strongly they hold their opinions. We show that our measurement is sensitive to all factors of polarization, a feat that is not achieved by the current state-of-the-art measures for polarization. We also show that the measure is able to unveil interesting insights in a number of real-world networks spanning from debates on Twitter to co-voting patterns in the U.S. House of Representatives.

This is the starting point of a promising research path. However, there are a number of caveats and limitations that can be amended in future works. In general, some caution is necessary when taking the δ_*G*,*o*_ estimations at face value. If one wants to talk about ideological polarization at an entire nation’s level, then they cannot rely on social media data like we do here. The social networks used here are a sample of the entire structure, and even if they considered the entirety of Twitter, it would still be a nonrepresentative sample of the population.

If the first caveat focuses on how *G* is built, then we also need caution when it comes to how *o* is estimated. δ_*G*,*o*_ scores are not compatible across networks if the ways to estimate *o* in different networks vary, as is the case between the Twitter and the U.S. House of Representatives networks.

In addition, we focus mainly on measuring and summarizing the opinion and structural component and their interplay in a single, consistent measure. Another relevant question for future work may be to determine how much each component contributes to the overall level of polarization in a network. In section S8, we show how to estimate the opinion component and the structural component on their own, as well as the strength of their correlation. A decomposition of δ_*G*,*o*_ into individual components might be interesting to understand how their importance has developed over time and to design evidence-based strategies that help reduce polarization on social media.

Another limitation is that δ_*G*,*o*_ is only apt at describing ideological polarization, that is the extent to which opinions get farther away toward extremism and people with different opinions tend to isolate from each other. Affective polarization, which pertains to how people with different opinions interact with each other (*11*), is also of great interest as it is the one truly affecting the quality of online discourse. One way that we could approach affective polarization is via network covariance (*52*) and/or correlations (*63*), since affective polarization should manifest as a correlation on the edges. Specifically, one would look whether the sentiment of a relationship is correlated with the opinion difference between the two individuals. These two approaches share commonalities with our δ_*G*,*o*_ measure; for instance, they all rely on effective resistance, showing how future work can expect to develop a coherent framework able to describe both ideological and affective polarization in consistent and comparable terms.

A further limitation, common in the literature, is that our measure assumes that people organize themselves in a 1D opinion space with only two poles. This describes somewhat well the U.S. political environment and debates with a clear “for” and “against” position. However, it has two drawbacks.

First, it is grossly underpowered for a multipole scenario such as the multiparty political systems common in many European countries. Multiple parties does not necessarily imply that there is a corresponding ideology dimension per party; nevertheless, creating a measure able to capture multiple ideological scales at the same time could be useful to avoid flattening everything on a two-pole system. There are some polarization-related studies for multiparty systems (*64*, *65*), but they do not quite capture the objective of this paper: estimating a single numeric score for a given *G*-*o* pair. Instead, they return a much more complex output describing the likelihood of two nodes to connect given their characteristics, data that might be unavailable. We can explore dimensionality reduction techniques to allow δ_*G*,*o*_ to tackle a scenario with multiple different opinions at the same time rather than just two. We outline one suggestion in section S6.

Second, by analyzing a debate at a time, we disregard the role of ideological consistency (*13*, *66*–*69*). We can expect, e.g., a person in favor of Obamacare to also be in favor of gun control and abortion rights. There are two ways to tackle ideological consistency. The first would be to use a multilayer network, in which each layer is a debate. Then, one can apply the multilayer version of δ_*G*,*o*_ (*70*) and get a polarization score that can be strengthened or weakened depending on the level of ideological consistency. Alternatively, one can calculate the network correlation between the different opinions of the individuals (*63*) to understand how consistent they are.

Our measure of polarization shares a drawback with all other data-driven approaches to polarization: If the data estimating the opinion of the individuals are inaccurate, the measure will provide inaccurate results. However, in section S7, we show how it is possible find upper and lower bounds of a polarization estimate if one knows how uncertain the opinion measurements are.

Other limitations involve the limited scalability of our approach, which is relatively memory-hungry and thus unable to tackle networks with millions of nodes. We plan to fix this issue in future work by using Laplacian solvers (*71*, *72*). We can also work on building a better intuition for the units of our measure and devise a way to normalize δ_*G*,*o*_ so that it takes values between, say, 0 and 1.

## ANALYTICAL APPROACH

### Interpretation of δ_*G*,*o*_

A convenient mental image to aid the interpretation of δ_*G*,*o*_ is the percolation of the opinions in a network, which can be modeled using discrete heat diffusion techniques. We can consider *o* as the temperature reading (opinion) of ∣*V*∣ thermometers, each located in a node. δ_*G*,*o*_ is directly proportional to the (square root of the) time it takes for heat to diffuse across the network and bring it to equilibrium.

**Fig. 9.**
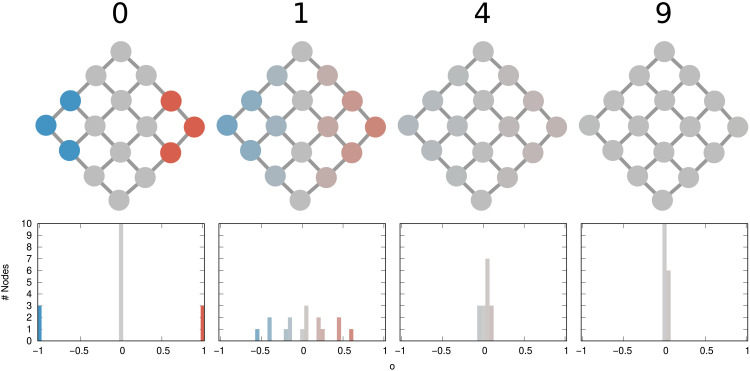
Polarization as heat diffusion. The graphs (second row) represent the status of the network at each time increment (first row). The histograms (third row) show the distribution of *o* values on the graph. Values in *o* span from −1 (blue) to +1 (red) passing via 0 (gray).

Figure 9 shows a graphical depiction of the diffusion process on a grid graph. The starting condition has some nodes in opposite corners at temperature −1 and +1. The polarization of this initial condition is δ_*G*,*o*_ ∼ 2.95, and we therefore expect it to take between 8 and 9 units of time for the system to converge to the average temperature (opinion), which is what we see if we run the simulation in the figure. For the simulation, we solve the discrete heat equation $\frac{do}{dt}=-Lo$ to find the solution at each time *t* (*73*).

The relation between $\delta_{G,o}^{2}$ and time to convergence is not always as direct as in this example, but in general, we find that the polarization δ_*G*,*o*_ is directly proportional to the time it takes to reach equilibrium, defined as the time *t* when the SD of the opinion vector *o*(*t*) goes below some fixed low ϵ value. We confirm this in section S4 by repeating the diffusion experiment on many input pairs *G*, *o*. In section S5, we further show how δ_*G*,*o*_ can be interpreted as a network version of the covariance between the *o*^+^ and *o*^−^ vectors.

### δ_*G*,*o*_ and effective resistance

δ_*G*,*o*_ also has a direct relationship with “effective resistance,” which is a robust way to measure distances between two nodes in a network and reflects the “effective number of steps” between two nodes (*58*). The effective resistance between two nodes *i* and *j* is denoted by ω*_ij_* and defined as

$$\omega_{ij}:={\left(e_{i}-e_{j}\right)}^{T}L^{†}(e_{i}-e_{j})$$

where *e_i_* is a vector with 1 at the *i*th entry and zeros otherwise. The effective resistance is proportional to the average time it takes for a random walker to go from node *i* to node *j* and then back again to *i*. In other words, this tells us how easy it is to traverse the network and move from one node to another and back. Compared to the shortest path distance, which measures the length of the shortest path between two nodes, the effective resistance takes into account the paths of all lengths and how they are interconnected.

To see why our measure of polarization is related to the effective resistance, let us consider a special case. Suppose that *o*^+^ and *o*^−^ are concentrated in two nodes, *i* and *j*, respectively, and zero otherwise. The polarization is then equal to

$$\delta_{G,o}=\sqrt{{\left(o_{i}^{+}-o_{j}^{-}\right)}^{T}L^{†}(o_{i}^{+}-o_{j}^{-})}=\sqrt{\omega_{ij}}$$

In other words, the larger the effective resistance between *i* and *j*, the larger the measured polarization.

This interpretation in terms of effective resistances also holds for balanced opinion distributions. For a balanced opinion distribution, we assume that the total sum of the positive opinions equals the sum of the negative opinions, e.g., $\sum \;o_{i}^{+}=\sum \;o_{i}^{-}=1$ and ∑ ‍ *o_i_* = 0. This can be trivially achieved by normalizing *o*^+^ and *o*^−^ with their sums. Then, we make use of the fact that for any zero-sum vector *x*, with $\sum_{i}$ ‍ *x_i_* = 0, the pseudo-inverse Laplacian product can be written in terms of the effective resistances as $\sum_{i,j}\;x_{i}{\left(L^{†}\right)}_{ij}x_{j}=-\frac{1}{2}\sum_{i,j}\;x_{i}\omega_{ij}x_{j}$; this follows from the definition of the effective resistance. Then

$$\begin{array}{rl} \delta_{G,o}= & \sqrt{{\left(o^{+}-o^{-}\right)}^{T}L^{†}(o^{+}-o^{-})}\\ = & \sqrt{\sum_{i,j}(o_{i}^{+}-o_{i}^{-}){\left(L^{†}\right)}_{ij}(o_{j}^{+}-o_{j}^{-})}\\ = & \sqrt{-\frac{1}{2}\sum_{i,j}(o_{i}^{+}-o_{i}^{-})\omega_{ij}(o_{j}^{+}-o_{j}^{-})}\\ = & \sqrt{\sum_{i,j}o_{i}^{+}o_{j}^{-}\omega_{ij}-\frac{1}{2}(\sum_{i,j}o_{i}^{+}o_{j}^{+}\omega_{ij}+\sum_{i,j}o_{i}^{-}o_{j}^{-}\omega_{ij})} \end{array}$$

Since all values $o_{i}^{+}$ are positive and sum to one, we can interpret this value as the probability of sampling a random individual *X*^+^ with a positive opinion. The probability of each individual is proportional to how extreme their opinion is, which is $\mathrm{Pr}[X^{+}=i]=o_{i}^{+}$.

For instance, Alice and Bob are both in favor of gun control, so *o*_A_, *o*_B_ > 0, but Alice is “twice as extreme” in her opinion as Bob and thus *o*_A_ = 2*o*_B_. When we select a random individual (*X*^+^) in favor of gun control, we will select Alice twice as likely as Bob since we get Pr[*X*^+^ = *A*] = 2Pr [*X*^+^ = *B*]. Similarly, we can consider a random individual *X*^−^ with a negative opinion based on the values $o_{i}^{-}$.

We can now formulate a probabilistic interpretation of the polarization δ_*G*,*o*_

$$\delta_{G,o}=\sqrt{E[\omega_{X^{+}X^{-}}]-\frac{1}{2}E[\omega_{X^{+}X^{+}}+\omega_{X^{-}X^{-}}]}$$

where the expectation operator E runs over the distribution over independent random variables *X*^+^ and *X*^−^. This formula has the following interpretation: δ_*G*,*o*_ measures the degree to which two individuals with conflicting opinions are more separated than two individuals with agreeing opinions. The polarization is thus the difference in distance between pairs of conflicting individuals and pairs of agreeing individuals, where individuals are selected according to the strength of their conviction. This shows that polarization is a relative measure that compares conflicting individuals with agreeing individuals.

This expression shows a possible generalization of δ_*G*,*o*_: If we have any notion of distance *d* between the nodes of a graph, then we can define a polarization score as

$$\delta_{G,o}=\sqrt{E[d(X^{+},X^{-})]-\frac{1}{2}E[d(X^{+},X^{+})+d(X^{-},X^{-})]}$$

This distance *d* could, for instance, be the shortest path distance between nodes in a network, the physical distance between individuals, or the travel time between locations.

### Computational complexity of δ_*G*,*o*_

If one estimates δ_*G*,*o*_’s formula naively, as we do here, the most expensive part of the framework is the calculation of *L*^†^, the pseudo-inverse of the Laplacian. This requires to solve the singular value decomposition problem for *L*. The cost is cubic, meaning that the algorithm can scale in the worst case as o(∣*V*∣^3^), and hence, it is inapplicable for networks larger than around 10^4^ nodes. However, we do not need to explicitly calculate *L*^†^ to calculate δ_*G*,*o*_. We can use Laplacian solvers (*71*, *72*), which can calculate the *L*^†^(*o*^+^ − *o*^−^) portion of δ_*G*,*o*_ in near-linear time. The complexity would then be o(∣*V*∣*^n^*), with 1 < *n* < 2, allowing the method to scale to much larger networks.

### Synthetic data generation

For the experiments showing the intuition and motivation of δ_*G*,*o*_, we rely on the generation of synthetic graphs *G* and opinion vectors *o*. Each *G* is generated using a simple SBM (*74*). To generate an SBM, one needs to specify the number of nodes ∣*V*∣, which we always set to 1000. The second ingredient is the assignment of nodes to communities. In our case, we create eight communities, each of the same size (125 nodes). The final two parameters are *p*_in_ and *p*_out_, which regulate the probability of two nodes in the same community (*p*_in_) or in different communities (*p*_out_) to connect to each other.

Each *o* is generated starting from a normal distribution of 500 values centered on 0 with an SD of 0.2. Then, in Fig. 3, we progressively create more and more polarization in the opinion distribution by shifting the average μ from 0 until 0.8, in 0.2 increments. We replace each value *o_x_* higher than 1 as follows: *o_x_* = 1 − (*o_x_* − 1). This ensures that all *o* values are lower than or equal to 1. Last, we set *o* = (*o*_0_, …, *o*_500_, − *o*_0_, …, − *o*_500_) and sort it, making it symmetric around 0 and of length 1000. Each community gets a continuous portion of *o*, ensuring opinion homophily inside the community.

For Fig. 3 we fix *p*_in_ = *p*_out_ = 0.0085. When *p*_in_ = *p*_out_, an SBM is equivalent to a plain random *G*_*n*,*p*_ graph (*75*). In a *G*_*n*,*p*_, each pair of nodes has the same probability of being connected, regardless of the community affiliations of the two nodes, and thus, there are no communities. For Fig. 4 we progressively decrease *p*_out_ from 0.0085 to 0.0003, correspondingly increasing *p*_in_ to keep the expected number of edges constant.

For Fig. 5, we set *p*_out_ = 0 between the nodes belonging to specific pairs of communities, increasing the other *p*_in_ and *p*_out_ accordingly to maintain the same expected number of edges. Specifically, we only keep connections between communities belonging to neighboring portions of the opinion spectrum *o*.

### Data collection

The gun control, abortion, and Obamacare networks were collected from Twitter. In all three cases, we retrieve the tweets related to each topic. To do so, we use the tweet ids provided by previous works (*34*), which follow the procedure outlined in the literature (*76*). From the tweets, we obtain a list of users involved in the debate. We create the network by collecting the 5000 most recent followers of each users, a cap that is imposed by Twitter’s rate limits.

We estimate the opinion of each user by looking at the URLs that they share. Each domain has an opinion score between −1 and +1, with the data coming from the fact-checking website https://mediabiasfactcheck.com/. The scores are provided directly by the website, placing each news source in a continuous −1 to +1 interval. The user’s opinion is the average of all the URLs that they have shared. This procedure is in line with the standard practice in the literature (*34*).

These networks have been used for many studies in the past (*38*), but there might be differences in their topologies because of the dynamic nature of Twitter. The original data source only provides tweet ids, not their content and no network information, as per Twitter’s terms of use. As a result, we need to recollect tweets and relationships that were established when the debates took place between 2015 and 2016. In the meantime, people might delete tweets, resulting in a different estimation of *o* because the relative frequency of URLs shared by a user changes. Moreover, users might follow/unfollow other users or even delete their account entirely, changing the edge and node sets of *G*.

We follow the literature in using only tweets that link to (at least) one of the URLs with a known opinion score. The dataset contains only users that have tweeted at least five times on either topic.

We apply the same procedure to generate the U.S. debate Twitter datasets, by collecting the networks about the second presidential debate, the vice presidential debate, and the election day, for the 2020 election. This is based on tweet ids collected by the George Washington University (*77*). In addition, in this case, we only use tweets that link to (at least) one of the URLs with a known opinion score. Different from above, we only consider users that have tweeted at least three times (and not five).

For the U.S. House of Representatives network, we collect roll call vote data from Voteview.com (*60*). We connect two congressmen from the U.S. House of Representatives following the procedure obtained from the literature (*78*), omitting votes from the Senate, as they would create a disconnected component in the network.

In practice, we connect nodes if the two members agree with one another on a vote more often than a specific Congress-dependent threshold. The threshold value is the number of agreements in a specific Congress where the pair of members is more likely to be from the same party than from opposing parties.

For most of the history of the U.S. House of Representatives, one could find a substantial number of cross-party agreements, leading to well-connected communities of Democrats and Republicans. This has stopped being the case from the 98th Congress, although the two communities are still part of a single connected component (otherwise, we could not apply δ_*G*,*o*_). Note that, with this procedure, a few nodes are isolated as they did not participate in enough votes to receive a connection, and thus, they are dropped from the networks.

## Supplementary Materials

### This PDF file includes:

Sections S1 to S9 Figs. S1 to S10 Table S1 to S6 References
