# Supplementary material for: Quantifying ideological polarization on a network using generalized Euclidean distance
Source: Sci Adv. 2023 Mar 1;9(9):eabq2044. doi: 10.1126/sciadv.abq2044 (PMC9977176; doi:10.1126/sciadv.abq2044)
Supplement: Supplementary file 1 — Sections S1 to S9 Figs. S1 to S10 Table S1 to S6 References [file sciadv.abq2044_sm.pdf]

Science Advances
AAAS

Supplementary Materials for

Quantifying ideological polarization on a network using generalized Euclidean distance

Marilena Hohmann *et al.*

Corresponding author: Michele Coscia, mcos@itu.dk

Sci. Adv. **9**, eabq2044 (2023)
DOI: 10.1126/sciadv.abq2044

The PDF file includes:

Sections S1 to S9
Figs. S1 to S10
Table S1 to S6
References

Other Supplementary Material for this manuscript includes the following:

File S1

1 Alternative Measures

1.1 Assortativity

Assortativity is a way to quantify the extent to which nodes with similar values connect to each other (*31*). The specific formula we use to estimate assortativity is the one developed by Newman (*30*).

This measure is calculated by creating a matrix e. Each entry e_{xy} in the matrix contains the fraction of the edges connecting two nodes in the network with values x and y of the measure of interest. This can be considered as a probability of the edge existing, implying that $\sum_{xy} e_{xy} = 1$.

For simplicity, we also record the fraction of edges originating from a node with value x ($\sum_y e_{xy} = a_x$) and the fraction of edges ending in a node with value y ($\sum_x e_{xy} = b_y$). If a network is undirected and unipartite, then $a_x = b_y$.

The assortativity is thus the Pearson correlation coefficient of x and y, which is:

$$\rho_{G,o} = \frac{\sum_{xy} xy(e_{xy} - a_x b_y)}{\sigma_a \sigma_b},$$

where σ_a and σ_b are the standard deviations of the distributions a_x and b_y. In this formulation, one needs the topology of G and the node values from the opinion vector o to build the matrix e (and, as a consequence, a_x and b_y).

Just like the Pearson correlation coefficient, this measure ranges from -1 (perfect disassortativity) to $+1$ (perfect assortativity).

It is not hard to see that assortativity cannot capture changes in the opinion component as defined and shown in the main paper: Suppose we have a vector of opinions o. Any linear change in o will generate the exact same assortativity value, provided that the values do not go beyond the $[-1, +1]$ interval. Halving all o values will generate the same assortativity value, even though this should be recognized as a lower polarization.

Assortativity also gives diminishing returns in capturing the structural component. It is linearly related to p_{out}, the probability of nodes in a community to connect outside their community, but linear changes in p_{out} have little effect for high p_{out} and large effects for low p_{out} – as Figure S1 shows. Networks that score the medium assortativity value ($\rho_{G,o} = 0.5$, we show a network with this value in the callout in the middle) are hardly distinguishable from networks scoring the minimum value ($\rho_{G,o} = 0.0$, we show an example of a network in the callout on the left). The regime in which networks have well-defined communities occupies a small fraction of the assortativity's domain.

In summary, assortativity can capture the alignment of opinions with the network struc-

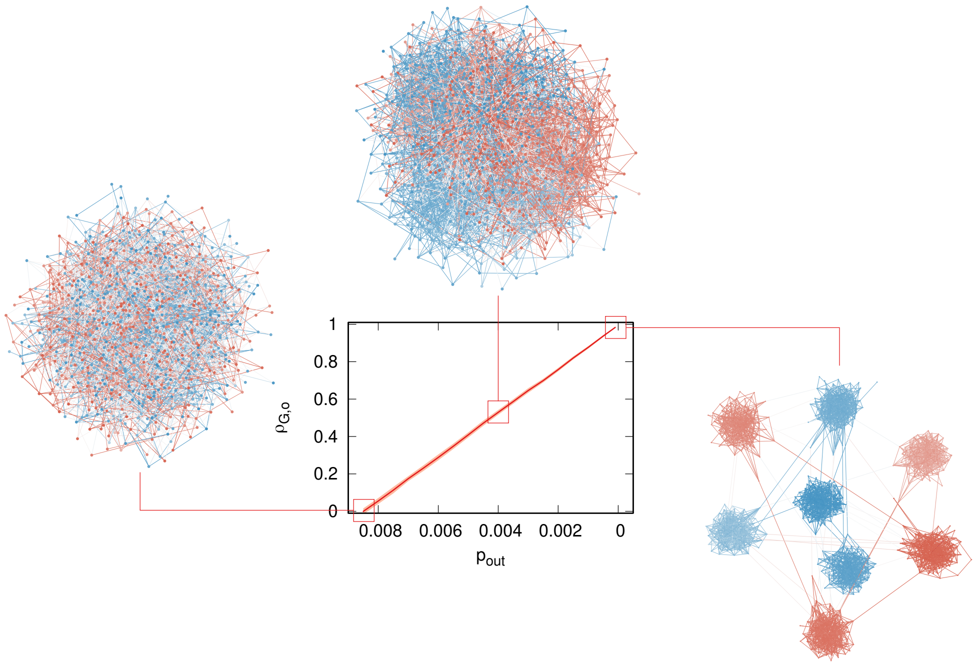

Figure S1: **The sensitivity of assortativity to the structural component**. $\rho_{G,o}$ values (y axis) for increasing levels of structural separation, i.e., decreasing p_{out} (x axis). The insets shows examples of networks at a given value of $\rho_{G,o}$, specifically (left to right): 0, 0.5, 1.

ture although it progressively loses sensitivity as we have better-defined communities, and it is wholly insensitive to the distribution of opinions.

1.2 Random Walk Controversy

Random Walk Controversy (RWC_G) is a measure that assumes the network can be partitioned into two opposing communities and then estimates the probability of a random walker to be able to transition from one community to another (*37*). The first operation is to bisect the graph into two communities. In the original paper, the authors use the METIS algorithm (*79*), although one could use any community discovery algorithm that can take the number of desired communities as input and that finds communities using a comparable definition as the one used by METIS (*80*).

Let us assume that we now have two communities C_1 and C_2, which are disjoint sets of

nodes ($C_1 \cap C_2 = \emptyset$) that cover the entire node set of G ($C_1 \cup C_2 = V$). RWC_G simulates a number of random walks – by default 10 times the number of nodes in the network – and records four probabilities: p_{C_1,C_2}, p_{C_2,C_1}, p_{C_1,C_1}, and p_{C_2,C_2}. In general, $p_{x,y}$ records the probability for a random walker to start in partition x and end in partition y. Half of the walkers start from C_1 and half from C_2. The walk terminates when it visits any node from a 10% random sample (from either side). Then:

$$RWC_G = p_{C_1,C_1} p_{C_2,C_2} - p_{C_1,C_2} p_{C_2,C_1}.$$

This measure is equal to $+1$ when all random walkers stay in their starting community, and equal to -1 when all random walkers end in the opposite community. A few things should be noted here.

First, the bisection into communities can be largely suboptimal because it is independent from the opinion vector o. If there are no clear-cut communities aligning themselves with o, then RWC_G's estimation is necessarily wrong. One could skip the community discovery and assign nodes to two communities according to whether their values in o are above or below 0. In either case, the actual distribution of o's values is irrelevant (it does not matter whether o values cluster tightly around 0 or at the extremes), and thus this measure cannot capture the opinion component by design, as we show in the main paper.

Second, the measure is not deterministic because it relies on a randomized simulation. One could fix this issue by deciding a fixed random walk length l, then take A^l – with A being the stochastic adjacency matrix of G. The result is the probability of a random walk starting from any node to reach any other node in l steps. Then one could estimate RWC_G with the formula above. In this case, RWC_G would be exact and deterministic. However, we do not perform this correction to stick with the original definition of RWC_G, noting that one could get different RWC_G values even with the same G and o.

Finally, in the original paper (*37*), the authors define multiple measures of polarization. Specifically, they also introduce versions using a random walk with restart, betweenness centrality, a 2D node embedding, Boundary Connectivity (*36*), and Dipole Moment (*26*). We do not investigate these variants as they are either highly correlated with RWC_G, or suffering from the same drawbacks about not considering the opinion distribution o.

1.3 Average Neighbor Opinion

In this approach, we estimate polarization by creating a density plot of opinions (*33*). For each node i in the network, we record its value o_i on the x axis. Then, the y axis value is derived from i's neighbor set N_i. Specifically, it is its average $\sum_{j \in N_i} o_j / |N_i|$.

The resulting density plot is then generated with a standard Kernel Density Estimation. A network is polarized if most of the density of points is concentrated among the top right and/or bottom left corners, implying that only nodes with similar values connect to each other.

As we show in the main paper, the issue with this approach is that it looks only at immediate neighbors, whereas any mesoscale organization of the network is not captured.

1.4 Influenced Set Opinion

This approach also estimates polarization graphically (*34*). Each node is considered in turn as the origin of a Susceptible-Infected-Recovered (SIR) epidemic event. In SIR, nodes start in a Susceptible state. They transition to Infected with a certain rate β when one or more of their neighbors are Infected. Finally, there is a recovery rate γ, which makes them transition to the Recovered state (*81*). Recovered nodes cannot be infected anymore. The process ends when all nodes are either in the Susceptible or Recovered state, and no more propagation can happen. In addition to epidemics, the SIR process can also model the spread of information or rumors on a network.

Say that R_i is the set of nodes in the Recovered state after the rumor propagates from node i. We can calculate their average opinion as $\sum_{j \in R_i} o_j/|R_i|$. Thus, this approach is exactly the same as the one we describe in the previous section, with the difference that we look at R_i (the set of infected nodes) rather than N_i (the direct neighbors of i).

Finally, rather than looking at the density plot, we bin all origin nodes according to their opinion value and show the distribution of the average influenced set nodes for each bin. A polarized network will show boxplots clustering on the top right and bottom left quadrants.

This can fix some of the conceptual problems of using the average opinion of the neighbours N_i, since R_i contains nodes that are not directly connected to i. However, a few more conceptual issues arise.

First, the β and γ parameters regulating the SIR event – as well as the number of bins in the plot – are chosen somewhat arbitrarily. While the authors of (*34*) show that this does not significantly impact the results as long as they are chosen within reasonable bounds, instructions on which bounds are reasonable are absent and rely on judgment calls from the analyst.

Second, just like RWC_G, the process relies on a randomized simulation, and thus can result in different estimations even with the same G and o.

Finally, while replacing N_v with R_v should help in capturing the mesoscale organization of G, this does not seem to happen in practice. Our experiments in the main paper show that different variations in of the opinion-structural interplay generate indistinguishable boxplots.

2 Properties of $\delta_{G,o}$

2.1 Lack of Maximum

$\delta_{G,o}$ lacks a maximum value, i.e., its domain is $[0, +\infty]$. That is to say, given a G and an o we can always find a G' and o' pair such that $\delta_{G,o} < \delta_{G',o'}$. We prove this statement by looking at a simple graph structure: a chain graph. In a chain graph, nodes are placed on a one dimensional

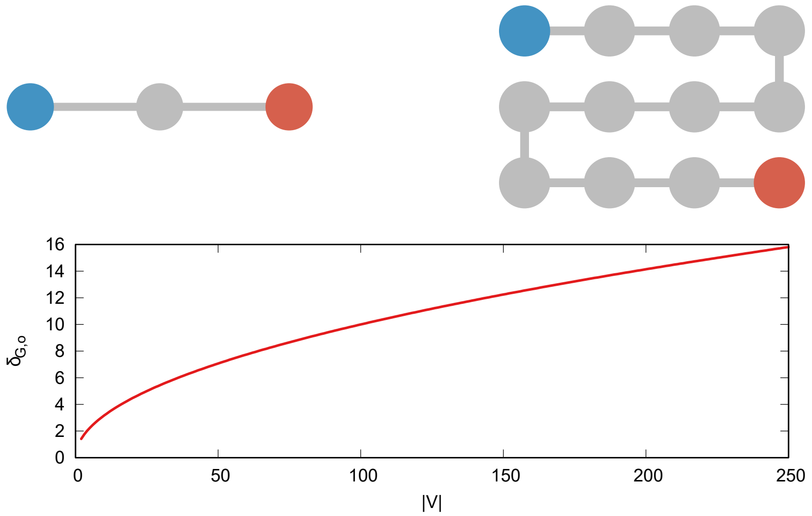

Figure S2: **The polarization values for growing chains**. (Top row) Examples of chains. The node color is proportional to the value in o (-1 blue, 0 gray, $+1$ red). (Bottom row) The value of $\delta_{G,o}$ (y axis) for a given number of nodes in the chain (x axis).

space and connected with their two closest neighbors with the exception of the two endpoints of the chain, which are only connected to their closest neighbor.

Then we generate a corresponding o vector. All entries in o are set to zero except the two entries for the endpoints of which one is set to -1 and the other to $+1$. We then grow the chain graphs by extending the chain as Figure S2 shows in the top row.

For each additional node in the chain, $\delta_{G,o}$ grows by a predictable amount. Specifically, $\delta_{G,o} = \sqrt{|E|}$ – see Figure S2 (bottom row). This is a known result in line with what was shown in the original paper defining the baseline node vector distance measure (*24*).

This is the reason why it is not possible to simply normalize $\delta_{G,o}$ to be defined between 0 and 1. There is not a well-defined maximum that can be used as a normalization factor.

One could narrow down the problem. Rather than finding the maximum of $\delta_{G,o}$ for any G, we could be looking for the maximum of $\delta_{G,o}$ for a given G, which implies finding the correct o vector. Unfortunately this is not a well-understood problem yet and thus it is not feasible to solve it here.

The possible opinion vectors must satisfy $o \in [-1,1]^{|V|}$, i.e., the only requirement is that $o_i \in [-1,1]$ for each i where $|V|$ is the number of nodes in the network. Hence, we may formalize the *maximum polarization problem* (MPP) as

$$\max_{o \in [-1,1]^{|V|}} \delta_{G,o} \tag{MPP}$$

Since the pseudoinverse Laplacian is positive semidefinite and the square root is monotone increasing on $\mathbb{R}^+$, we may simply square the objective function $\delta_{G,o} \to \delta^2_{G,o}$ and equivalently write MPP as:

$$\max_{o \in [-1,1]^{|V|}} o^T L^\dagger o$$

and then take the square root of the solution. As a first result, we can show that the opinion vector $o^\star$ that achieves the maximum polarization will necessarily have entries that are either $+1$ or -1. This implies that the domain of MPP can be changed from $[-1,1]^{|V|}$ to $\{-1,+1\}^{|V|}$, i.e., with $o_i = \pm 1$ for every node i.

Proposition 1 *The opinion vector $o^\star$ that solves MPP lies in $\{-1,+1\}^{|V|}$.*

Proof: Since both $L^\dagger$ and the domain for o are bounded, $\delta_{G,o}$ will be bounded from above by some finite number and thus at least a supremum exists. As the domain $[-1,1]^{|V|}$ is compact, this supremum is achieved by some $o^\star$, the optimal solution to MPP. There is no uniqueness, e.g., $\delta_{G,o^\star} = \delta_{G,-o^\star}$, but this is not required for the proof. Assume for contradiction that there is a node i for which $o^\star_i \in (-1,+1)$, i.e., not equal to ± 1.

Consider what happens if we increase or decrease the opinion of i, i.e., $o^\star_i \pm \epsilon$, with ϵ small enough such that this lies in $[-1,1]$. Then the node vector distance becomes:

$$(o^\star + \epsilon e_i)^T L^\dagger (o^\star + \epsilon e_i) = \delta^2_{G,o^\star} + \epsilon^2 e_i^T L^\dagger e_i + 2\epsilon e_i^T L^\dagger o^\star,$$

where e_i is the ith unit vector. Since $\epsilon^2 > 0$ and $L^\dagger$ is positive semidefinite with kernel $L^\dagger o = 0$ if and only if o is a constant vector (which e_i is not), the second term is positive. For the last term, we may choose the sign of ϵ such that the term becomes positive, i.e. $\text{sign}(\epsilon) = \text{sign}(e_i^T L^\dagger o^\star)$ then we find that

$$\delta_{G,o^\star+\epsilon e_i} > \delta_{G,o^\star}$$

But this is in contradiction with $o^\star$ being the maximum polarization vector, hence our assumption that a node i with $o_i \in (-1,1)$ exists must be false and this concludes the proof. □

Following this proposition, we can rewrite the MPP as:

$$\max_{o\in\{-1,1\}^{|V|}} o^T L^\dagger o,$$

i.e., where the optimization domain for o is now changed. We note that instead of choosing a vector o in this domain, we may equivalently choose two subsets o^+, o^- which then determine the vector o.

In this form, MPP is equivalent to the so-called *(weighted) MAX CUT problem* (*82*) where the weight signs are determined by the off-diagonal entries of $L^\dagger$. If the off-diagonal is non-positive (≤ 0), this problem is known to be NP hard. If the entries have mixed signs, as in the case of MPP, the problem complexity transitions from hard to easy, as described in (*82*). But this transition is not fully understood and we cannot be conclusive on the complexity of MPP in general.

2.2 Scale Invariance

$\delta_{G,o}$ is scale invariant, meaning that equivalent topologies will get the same score even if they have a different number of nodes. Of course, networks with a different number of nodes also

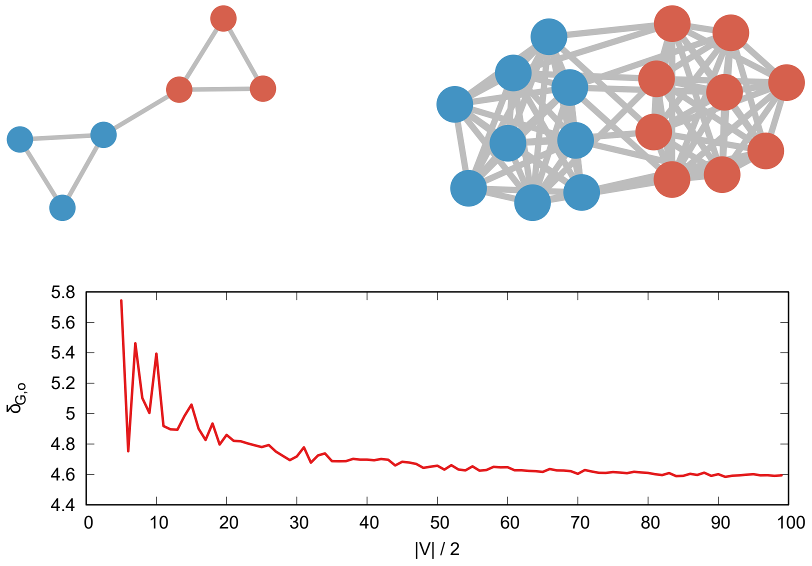

Figure S3: **The polarization values for growing connected cliques**. (Top row) Examples of connected cliques. The node color is proportional to the value in o (-1 blue, $+1$ red). (Bottom row) The value of $\delta_{G,o}$ (y axis) for a given number of nodes in the cliques (x axis).

have a slightly different topology, so the score is not exactly the same, but it will tend to the same limit.

To illustrate, let us consider a relatively simple topology. We have a network G with two cliques, connected by few edges between them. Specifically, the number of edges connecting the two cliques is 5% of the number of edges inside each clique. For instance, if the cliques contain 25 nodes, then there are 15 edges between them ($15 = 0.05 \times 25(25-1)/2$). The edges between the cliques are established randomly. The vector o assigns a value of -1 to all nodes in one of the two cliques, and $+1$ to the nodes of the other.

We can now grow the network by increasing the size of the two cliques one node at a time, while adding edges between the cliques to keep the density constant. Figure S3 shows examples of the generated graphs with their corresponding polarization score $\delta_{G,o}$. We can see that the scores tend to a finite limit. The larger the system, the closer the score gets to the limit, as the randomization of the connections between the cliques plays a smaller role in creating

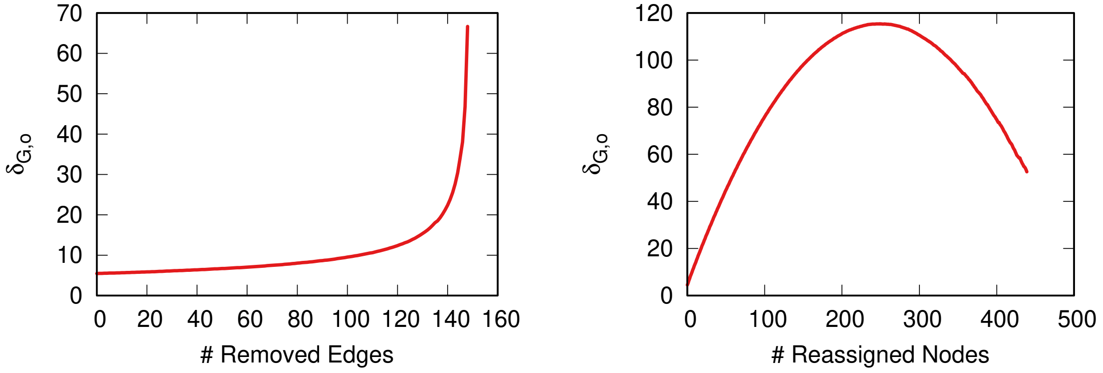

Figure S4: **The polarization values for a two-community system**. $\delta_{G,o}$ (y axis) as we modify the topology of the network. (Left) X axis records the number of inter-community edges removed. (Right) X axis records the number of nodes reassigned from the $+1$ to the -1 community.

fluctuations in the score.

On the other hand, if we modify the actual topology of the network, the score will change even if the scale – the number of nodes – remains constant. We illustrate this with two further simulations.

In the first simulation, we have two equally sized communities, each containing 250 nodes. One community has all positive random values in o while the other has negative random values. We build this with a stochastic blockmodel, ensuring that there are roughly 150 edges between the two communities. We then start removing these edges between the blocks one by one.

Intuitively, this should make the network more polarized, as it becomes harder and harder for an opinion to propagate from one community to the other. This is exactly what we see in the scores of $\delta_{G,o}$, as Figure S4 (left) shows. The first removed edges have little impact, and the impact of each removed edge grows exponentially as we get closer and closer to isolating the two communities.

In the second simulation, we also have two blocks, but we start from an unbalanced situation. One community has 495 nodes while the other only has 5. Every node in one community has

an opinion value of $+1$, while every node in the other community has an opinion value of -1. Then, we select one node at random from the large community and we move it to the small community. We remove all of its connections to its old community and we add connections to the new community at random, preserving its degree (if there are enough candidate neighbors in the community to do so). Its opinion value also flips to align with its new community.

Figure S4 (right) shows the effect. Initially, as we reassign the first nodes, $\delta_{G,o}$ grows. This is expected, since in the starting condition polarization is low as most people have the same opinion and connect to each other. Around the 250th reassignment, $\delta_{G,o}$ plateaus and then starts to decrease again. This shows how $\delta_{G,o}$ peaks when the conflicting communities are roughly the same size. As soon as one community starts getting dominant in size, this lowers the polarization of the system. The process of reassigning nodes stops at around 450 nodes because the network becomes disconnected as a result of randomly removing nodes.

2.3 Effect of Density & Fragmentation

$\delta_{G,o}$ is sensitive to density, because the more dense a network is, the easier it is for an individual to be exposed to a dissenting point of view. This sensitivity can be tested in two ways.

First, we can test it directly. We create a network of 1,680 nodes and divide it in two cliques. The probability of edges being established between the cliques is 5%, i.e., $p_{out} = 0.05$. Then we start removing edges at random from this network, rendering it progressively less dense. Figure S5 (left) shows the growth of $\delta_{G,o}$ as we remove more and more edges.

A second way for a network to become less dense is by fragmenting its community structure. A network with many smaller cliques is less dense than a network with few large ones, even when keeping the number of nodes and p_{out} constant. As before, $\delta_{G,o}$ should grow because people become more and more isolated and it is therefore harder to be exposed to both opposing but also conforming views.

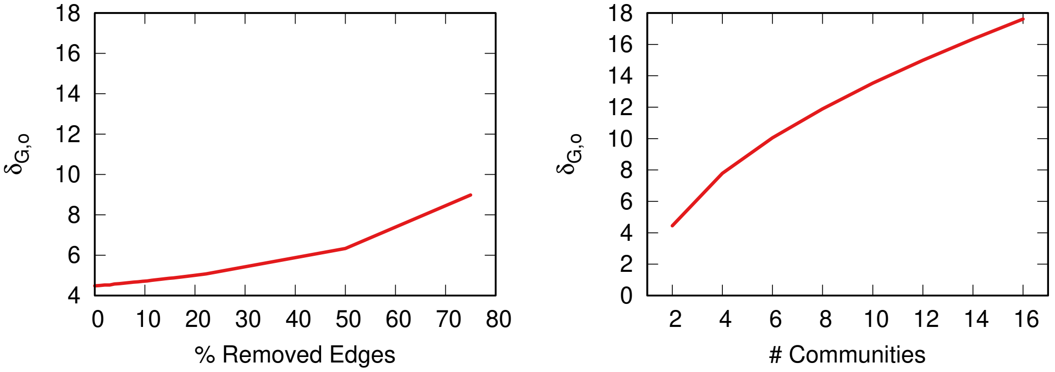

Figure S5: **The polarization values for increasingly sparse networks**. $\delta_{G,o}$ (y axis) as we: (left) remove a fraction of edges at random (x axis); or (right) fragment it into more communities (x axis).

To test this, we take our network of 1,680 nodes. We then divide it into a growing number of cliques, from 2 to 16. All nodes in each clique have an o value of either $+1$ or -1, and there is an equal number of cliques with either opinion value. We then connect cliques with $p_{out} = 0.05$. We only induce an even number of cliques to avoid having one opinion being over-represented in the leftover clique – 1,680 is the smallest number divisible by all even numbers between 2 to 16.

Figure S5 (right) shows the growth of $\delta_{G,o}$ as we increase the fragmentation in communities, which matches this intuition.

3 The Polarization Component Space

3.1 Main Results

To test our measure of polarization, we generate synthetic networks that vary along three different parameters and explore how $\delta_{G,o}$ changes along. In Figures 2 to 4 in the main text, one parameter is varied at a time with the other two fixed but here we consider the full extent of the parameter space.

We explore the three parameters as follows. The first parameter (μ) regulates the divergence

of opinions. Each side of the opinion vector o peaks at $\pm\mu$. Thus, if μ is 0.2, the left side of o peaks at -0.2 and the right side peaks at 0.2. For this reason, the higher the μ value, the more polarization there is, as Figure 2 in the main paper shows.

The second parameter (p_{out}) regulates the structural component by determining the probability of a node in a community to connect to a node in a different community – as we describe in the Materials and Methods section in the main paper. The lower p_{out}, the more polarization there is, as nodes get progressively more isolated from opinions in outside communities.

Finally, for the mesoscale interplay, we use the parameter n which indicates how many communities a community can link to. We pick possible neighboring communities among the n closest ones in the opinion spectrum o. For example, if $n = 7$, all communities can link to each other because with 8 communities there are only 7 possible neighbors. If $n = 1$, a community can only link to its most immediate neighbor. The lower the n value, the more polarization there is, because communities get progressively more isolated from potential connections with nodes in a different portion of the opinion vector o.

Table S1 shows all values of $\delta_{G,o}$ across the three parameters. It reports the average of 25 randomly initialized runs. The standard errors of these means are in the order of 1% of the mean value. This shows that these scores are stable and reliable as there are no wild fluctuations from small differences in random initialization. Table S1 shows a smooth transition across these parameters with no significant discontinuities. This supports our claim in the main paper that $\delta_{G,o}$ is sensitive to all these factors.

3.2 Necessary Conditions for Polarization

While the opinion and structural component – and their interplay – are all important to quantify polarization, there can be some degenerate cases in which one or two parameters out of three dominate the value of $\delta_{G,o}$. The most straightforward case is one in which there is no polariza-

μ	p_{out}	n 7	6	5	4	3	2	1
	0.0085	2.56	2.75	3.10	3.54	4.33	5.80	9.89
	0.0042	3.47	3.81	4.43	5.23	6.69	9.89	19.73
0.0	0.0024	4.55	5.01	5.81	7.00	9.13	13.93	28.91
	0.0012	6.38	7.09	8.14	9.93	13.48	20.48	43.16
	0.0006	9.20	10.41	11.82	14.45	19.16	30.73	66.46
	0.0003	13.22	14.70	17.21	20.71	28.68	44.81	100.27
	0.0085	3.56	3.95	4.41	5.03	6.11	8.23	14.40
	0.0042	4.94	5.42	6.25	7.38	9.54	14.10	28.20
0.2	0.0024	6.55	7.10	8.33	9.93	13.01	19.80	42.43
	0.0012	8.99	10.16	11.61	14.32	19.11	29.24	62.86
	0.0006	13.15	14.88	16.65	20.36	27.82	44.28	95.31
	0.0003	18.81	20.92	24.42	28.97	41.20	63.45	144.62
	0.0085	5.60	6.01	6.75	7.77	9.58	13.27	23.33
	0.0042	7.88	8.47	9.64	11.51	15.14	22.79	46.10
0.4	0.0024	10.30	11.07	12.82	15.48	20.76	31.93	67.58
	0.0012	14.46	15.66	18.15	22.32	30.24	47.19	101.40
	0.0006	20.95	23.19	25.94	32.18	43.92	70.57	153.19
	0.0003	29.88	32.43	38.52	45.72	65.51	102.47	232.88
	0.0085	7.86	8.33	9.23	10.66	13.39	18.43	32.27
	0.0042	11.08	11.73	13.26	15.84	21.04	31.88	64.33
0.6	0.0024	14.51	15.37	17.76	21.42	28.93	44.79	94.79
	0.0012	20.55	21.88	25.19	30.76	42.42	65.49	141.78
	0.0006	29.58	32.15	35.65	44.44	61.07	98.63	213.88
	0.0003	42.21	45.08	52.66	62.80	90.67	144.05	328.65
	0.0085	9.79	10.27	11.24	12.96	16.37	22.61	39.81
	0.0042	13.86	14.47	16.20	19.36	25.89	39.22	79.18
0.8	0.0024	18.20	19.08	21.76	26.11	35.44	54.83	116.96
	0.0012	25.70	26.99	30.65	37.53	51.98	80.82	174.90
	0.0006	37.00	39.70	43.70	54.28	74.59	121.25	264.56
	0.0003	52.93	55.57	64.44	76.14	110.66	176.67	405.67

Table S1: **The evolution of polarization scores across all parameters**. Each cell reports the value of $\delta_{G,o}$ for different values of the opinion component (μ, row groups), the structural component (p_{out}, rows), and the opinion-structural mesolevel interplay (n, columns). Each cell background is colored proportionally to the value of $\delta_{G,o}$, from low (bright red) to high (dark red).

tion due to the structural component. This is the case in which the network contains only one node. As soon as we have two nodes we can measure distances between individuals and, as a consequence, we have a non-zero contribution of the structural component to polarization and $\delta_{G,o}$.

The case of no polarization from the opinion component is more interesting. If everyone has the same opinion, polarization should be zero, independent of the social structure. This is the case for $\delta_{G,o}$ by definition, because if o is constant, then $o^+ - o^-$ is zero and the whole formula evaluates to zero. We can show this by creating a network with extreme structural separation – two large cliques connected by few edges – and by drawing o from a normal distribution with decreasing standard deviation.

Figure S6 shows what happens as we approach zero opinion divergence: $\delta_{G,o}$ naturally captures the decrease in diversity of opinions. Both assortativity and RWC_G are blind to those changes and still estimate a constant polarization value even for insignificant differences of opinion. The average neighbor opinion plot could capture the differences, provided the KDE is initialized with the proper parameters – here we fail to see a difference between the last two cases only because we have too large bandwidth. The average influenced set opinion can detect a lack of opinion divergence.

3.3 Secondary Polarization Components

It is possible to consider other aspects as potential components of polarization. For instance, in Figure 1 in the main paper, we move from a random $G_{n,p}$ graph with random opinion assignments to a graph with communities that have assortative opinions. In other words, we make communities whose nodes all occupy a contiguous portion of the opinion spectrum: a node with a given opinion will be embedded in a community whose nodes have a similar opinion to them.

In doing this, we are skipping over a potential polarization component: what happens if we

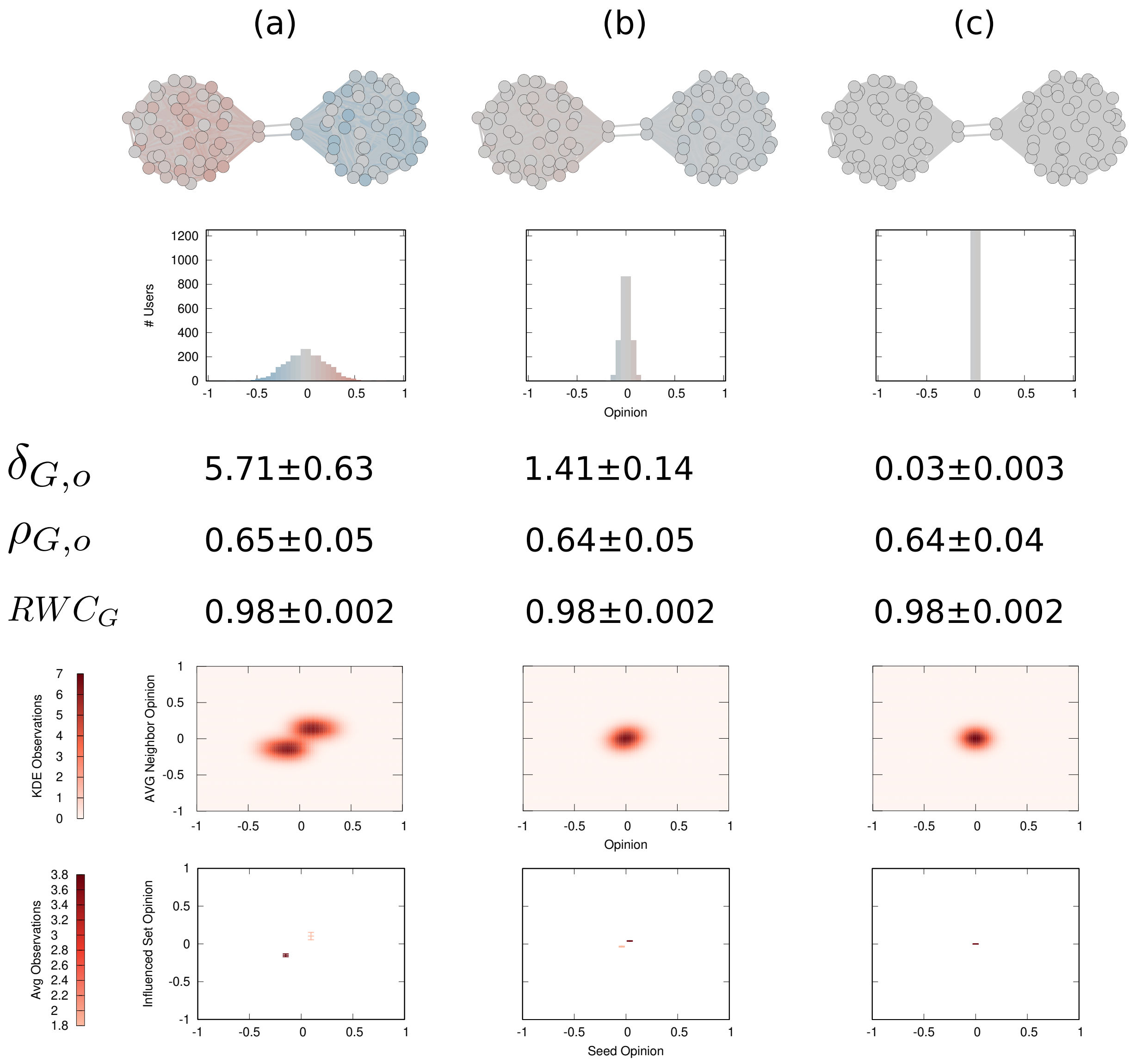

Figure S6: **Polarization for vanishing opinion divergence**. Each row shows (top to bottom): the network structure; the opinion distribution; the average values over 25 runs of $\delta_{G,o}$, $\rho_{G,o}$, and RWC_G with their standard deviations; the density maps of opinions (x axis) and average neighbor opinion (y axis); boxplots of seed opinion (x axis) and average opinion of the influenced set after a SIR propagation (y axis). The opinion distribution is a normal distribution with average zero and standard deviation of (a) 0.2, (b) 0.05, (c) 0.001.

have structural communities, but opinions are still randomly distributed within each community? In such a scenario, having a given opinion tells a node nothing about their companions in

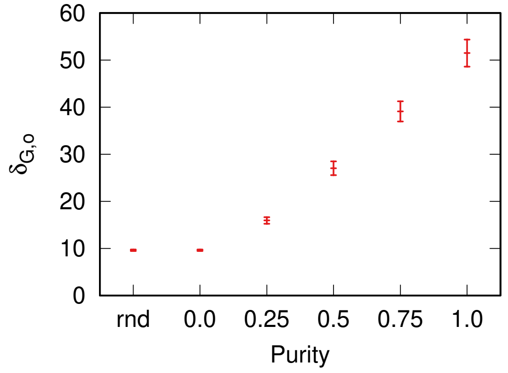

Figure S7: **Polarization and opinion purity**. The average and standard deviation value of $\delta_{G,o}$ (y axis) across 50 independent runs for a random graph and different levels of purity in a graph with strong communities.

the same community.

The reason we do not consider this as a polarization component is because polarization would not change in this extreme scenario. If opinions are distributed randomly, it does not matter whether there are communities or whether the graph is random, because each community in isolation could be considered as a sort of random graph.

To illustrate, we create a graph with 8 communities using an SBM and random o assignment with $\mu = 0.8$ (high opinion divergence) and $p_{out} = 0.0003$ (which should imply high structural separation). After 50 independent runs, we observe $\delta_{G,o} = 9.63 \pm 0.16$. For each run, we also create a randomized version of the graph by performing enough double edge swaps to render the graph effectively random. This random graph has an indistinguishable polarization score: $\delta_{G,o} = 9.64 \pm 0.17$. We do edge swaps rather than generating an alternative random graph because this way we can ensure that the graphs have the exact same number of edges.

The real factor that induces polarization in the system is a community's purity – how well-aligned communities are with o scores. If purity is 1, all nodes in all communities share similar opinion scores. If purity is 0, opinion scores are random. Figure S7 shows how $\delta_{G,o}$ varies as we increase the purity of our communities. This empirical result matches with what we would

expect theoretically: in the low purity networks, everyone is exposed to different views and the polarization score should be low.

Since one cannot have pure communities if there are no communities, we prefer using p_{out} to discuss the structural component of polarization rather than purity.

4 Interpretation of the Units

In the Materials and Methods section in the main paper, we show the relationship between $\delta_{G,o}$ and effective resistance and heat diffusion processes. Here, we show what happens if some of the assumptions we made do not hold, and we provide additional information about these relationships.

4.1 $\delta_{G,o}$ and Effective Resistance in General

If the assumptions that $\sum o_i^+ = \sum o_i^- = 1$ do not hold, and it is not reasonable to normalize the vectors such that we can make them hold by construction, we can still arrive at a similar interpretation. Let $\bar{o} := \frac{1}{|V|} \sum o_i$ be the overall mean opinion. Then we say that two individuals agree if they are on the same "side" of $\bar{o}$ (both larger, or both smaller), and disagree if they are not. Then we assign to every node the variable y equal to $y_i = \frac{(o_i - \bar{o})}{\frac{1}{2} \sum |o_i - \bar{o}|}$, and we consider the positive and negative opinions y^+ and y^- as before. In other words, the variable y_i captures how far an opinion is from the mean, and whether it is larger or smaller than the mean. We now notice that for y^+ and y^- the following holds: by construction – i.e., by removing the mean – we know that the positive sum equals the negative sum, and by normalization with $Z := \frac{1}{2} \sum |o_i - \bar{o}|$, the sums equal one. We can thus define the random variables Y^+ and Y^- with distribution given by y^+ and y^- and find that, in general, the polarization can be interpreted as:

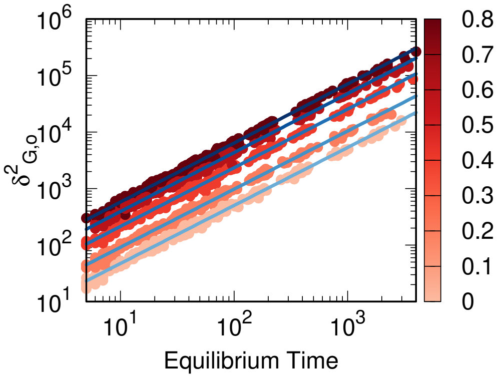

Figure S8: **The relationship between polarization score and heat diffusion equilibrium**. The squared $\delta_{G,o}$ value (y axis) against the unit of time in which heat diffusion reaches equilibrium (x axis). Color encodes the opinion divergence factor μ. The blue line shows the best fits calculated for each μ factor independently.

$$\delta_{G,o} = \left(\frac{1}{2}\sum |o_i - \bar{o}|\right) \sqrt{\mathbb{E}[\omega_{Y^+Y^-}] - \tfrac{1}{2}\mathbb{E}[\omega_{Y^+Y^+} + \omega_{Y^-Y^-}]},$$

so it is still the difference in distance between conflicting individuals and agreeing individuals, but in this case conflicting and agreeing is defined based on whether they are on the same side of the mean opinion or not; similarly the probability of an individual is proportional to their distance to the mean. Furthermore, the factor Z is included, which quantifies how far the opinions are, on average, from the mean opinion.

4.2 Heat Diffusion

In support of our interpretation of $\delta_{G,o}$ as the (square root of the) time it takes for heat to diffuse in the network, we calculate both values on the collections of SBMs used to generate Table S1. That is, we explore all possible values of opinion divergence μ, community interconnectedness p_{out}, and mesolevel organization n.

Figure S8 shows the result. We can see that the opinion divergence factor μ provides a constant multiplicative factor – if the opinion vectors are more extreme it takes a constant additional

amount of time to reach equilibrium. If we take that factor out, $\delta^2_{G,o}$ correlates almost perfectly with the amount of time required for o to have a standard deviation lower than $\epsilon = 2 \times 10^{-4}$. The choice of ϵ does not matter, provided it is a small value.

All the power laws we calculated independently for each μ value have an $R^2 \sim 0.996$, and an exponent approximately equal to 1, showing a linear relationship between $\delta^2_{G,o}$ and equilibrium time.

5 Relations to Network Covariance

As we show in the Materials and Methods section in the main paper, $\delta_{G,o}$ *de facto* measures the Generalized Euclidean distance (*24*) between two vectors o^+ and o^- on the nodes of a given graph G. We show here that this polarization measure can also be seen as a measure of covariance between the opposing opinion distributions, in the sense of (*52*). If V_+ are the nodes with positive opinion $o_i > 0$ and V_- the nodes with negative opinion, then we define the following joint distribution P between pairs of nodes

$$(P)_{ij} := \begin{cases} \frac{1}{2} o_i o_j \text{ if } i \in V_+ \text{ and } j \in V_-, \\ \frac{1}{2} o_i o_j \text{ if } i \in V_- \text{ and } j \in V_+, \\ 0 \text{ otherwise,} \end{cases}$$

which can also be written as a $|V| \times |V|$ matrix as:

$$P = \frac{1}{2} \left(o^+ o^{-T} + o^- o^{+T} \right).$$

where $o^{\pm}$ are the vectors with only positive (negative) opinions. This is a distribution which selects a random pair of nodes with opposing opinions, with probability of picking each node proportional to their opinion. So if $o_v = 2o_w$, then v is twice as likely to be picked as w. The covariance of this joint distribution, with respect to the effective resistance matrix $\Omega = (\omega_{ij})$, is

calculated according to (*52*) as

$$
\begin{aligned}
\mathrm{cov}_\omega(P) &= \frac{1}{2}\,\mathrm{tr}\left(\left(P\mathbf{1}\mathbf{1}^T P - P\right)\Omega\right) \qquad \text{(with } \mathbf{1} = (1,\ldots,1)\text{)}\\
&= \frac{1}{2}\mathbf{1}^T P\Omega P\mathbf{1} - \frac{1}{2}\,\mathrm{tr}\left(P\Omega\right)\\
&= \frac{1}{2}\left(\frac{1}{4}o^{+T}\Omega o^{+} + \frac{1}{4}o^{-T}\Omega o^{-} + \frac{1}{2}o^{+T}\Omega o^{-}\right) - \frac{1}{2}\left(o^{+T}\Omega o^{-}\right)\\
&= \frac{1}{2}\left(\frac{1}{4}o^{+T}\Omega o^{+} + \frac{1}{4}o^{-T}\Omega o^{-} - \frac{1}{2}o^{+T}\Omega o^{-}\right)\\
&= \frac{1}{8}\left(o^{+} - o^{-}\right)^T \Omega \left(o^{+} - o^{-}\right)\\
&= -\frac{1}{4}\left(o^{+} - o^{-}\right)^T L^{\dagger} \left(o^{+} - o^{-}\right) \qquad \text{(following the definition of effective resistance)}\\
&= -\frac{1}{4}\delta^2_{G,o}
\end{aligned}
$$

This shows that $\delta_{G,o}$ is proportional to minus the covariance of distribution P. Our polarization measure captures the covariance of a pair of nodes with opposing opinions – a high covariance signifies that they are close together in the network, while a small covariance indicates that they are far apart. This is in line with how our measure intends to quantify polarization.

6 Multidimensional Polarization

6.1 Formulation

As introduced, our polarization measure $\delta_{G,o}$ is limited to measuring the polarization that arises in a social network on a binary or unidimensional topic. Any individual can have one of two opinions $\{+, -\}$, with a given strength or conviction recorded in the vector o. While useful in the case of debates with {for, against} stances or in two-party political systems such as the United States, this approach cannot handle the case of multidimensional issues. Here, we briefly discuss how our binary measure can be extended to deal with these more complex cases.

Let us assume that any individual can support an opinion in a set of options $\{A_1, A_2, \ldots, A_k\}$ – these can for instance be k political parties – with a certain strength given by the vector o^{A_i}

that records for each individual how strongly they support opinion A_i. An overall measure of polarization can then be obtained as an average over all possible pairwise conflicts, as

$$\delta_{G,o} = \sqrt{\binom{k}{2}^{-1} \sum_{i=1}^{k} \sum_{j=i+1}^{k} (o^{A_i} - o^{A_j})^T L^{\dagger} (o^{A_i} - o^{A_j})}.$$

If all the opinion vectors are normalized, then the same derivation we use in the Methods section in the main paper can be used here, which means that this multidimensional polarization can intuitively still be interpreted as a measure of the difference in (effective resistance) distance between disagreeing and agreeing individuals. We note that other extensions to multidimensional measures might be possible, for instance assigning a different weight to the different pairwise conflicts.

6.2 Behavior

We can show that the multidimensional version of our measure behaves in the same way as the original measure across the various components and it is equally intuitive. To do so, we repeat our synthetic experiments. Rather than having two vectors o^+ and o^-, we now have eight different opinions since our synthetic networks contain eight communities. Each community i contains only nodes with nonzero values for opinion A_i and zero for all other opinions A_j. The opinion vectors are constructed with the same procedure outlined for the simple unidimensional case, with μ representing the mode value. We construct the network with the same algorithm, so also parameters p_{out} and n retain their meaning.

By running the experiments, we can create an equivalent of Table S1 for the multidimensional case. Table S2 is the result. We can see that the overall behavior of the multidimensional measure is the same as the unidimensional one. We can then conclude that the interpretation of the two measures is the same, and that we can apply our extension to the multidimensional case.

μ	p_{out}	n 7	6	5	4	3	2	1
	0.0085	1.24	1.25	1.27	1.34	1.39	1.54	2.09
	0.0042	1.60	1.60	1.69	1.79	1.91	2.40	4.07
0.0	0.0024	2.02	2.06	2.08	2.28	2.57	3.31	6.00
	0.0012	2.75	2.83	2.98	3.21	3.66	4.73	8.77
	0.0006	3.78	3.90	4.15	4.52	5.31	6.78	13.38
	0.0003	5.45	5.65	5.91	6.57	7.59	10.38	19.86
	0.0085	1.78	1.78	1.83	1.90	1.97	2.19	3.14
	0.0042	2.29	2.30	2.40	2.56	2.79	3.40	5.84
0.2	0.0024	2.88	2.97	3.00	3.29	3.81	4.71	8.62
	0.0012	3.91	4.02	4.27	4.54	5.21	6.97	13.16
	0.0006	5.63	5.61	6.08	6.59	7.66	10.12	19.64
	0.0003	7.87	8.24	8.58	9.63	11.14	15.34	29.61
	0.0085	2.80	2.81	2.86	3.00	3.15	3.57	5.25
	0.0042	3.70	3.81	3.93	4.28	4.69	5.84	10.20
0.4	0.0024	4.83	4.89	5.16	5.54	6.33	8.11	14.85
	0.0012	6.67	6.93	7.19	7.90	9.35	11.80	22.70
	0.0006	9.58	9.88	10.62	11.31	13.25	17.70	33.97
	0.0003	13.65	13.90	14.86	16.92	18.94	26.21	51.17
	0.0085	3.93	3.97	4.05	4.20	4.48	5.14	7.56
	0.0042	5.46	5.48	5.68	6.14	6.77	8.62	14.89
0.6	0.0024	7.01	7.19	7.50	8.12	9.38	11.97	22.21
	0.0012	10.01	10.22	10.58	11.55	13.49	17.56	33.84
	0.0006	14.10	14.45	15.30	16.51	19.62	25.88	50.81
	0.0003	20.14	20.86	21.93	24.53	28.28	38.41	75.07
	0.0085	4.89	4.94	5.04	5.24	5.60	6.50	9.69
	0.0042	6.85	6.88	7.21	7.73	8.66	11.10	19.19
0.8	0.0024	8.97	9.10	9.50	10.37	11.90	15.30	28.24
	0.0012	12.70	13.07	13.64	14.79	17.27	22.81	43.36
	0.0006	18.08	18.42	19.47	21.05	24.95	33.07	64.88
	0.0003	25.81	26.72	28.00	31.80	36.27	49.79	96.50

Table S2: **The evolution of multidimensional polarization scores across all parameters**. Same legend as Table S1.

The only difference is that the absolute values of the measure tend to be smaller. This might be due to the fact that the average of all pairwise conflicts decreases as we increase the number of opinions, as by construction some opinions are closer to each other. However, we leave this

investigation for future work, along with the consideration that taking the average of all pairwise conflicts might not necessarily be the best way to expand our measure.

7 Sensitivity to Measurement Errors

While we have assumed that the network G and opinion vector o are given as inputs from which we calculate the polarization, it is important to acknowledge that this data is at best an approximation of the real underlying opinions and social network connections. In particular, measurement errors and other inherent difficulties in obtaining and representing such complex data might mean that the available opinion vector o only records the opinions approximately. This can be modeled as an additive measurement error $o = \hat{o} + \epsilon$ where $\hat{o}$ is the real opinion vector and ϵ the error vector. If ϵ is large relative to the measurements, i.e., the errors are of the same order as the opinions (and uncorrelated), then there is no real hope for a measure that accurately represents the real polarization. If, on the other hand, the error vector is relatively small, we would hope that the polarization measure is a good approximation.

This is the case for our proposed polarization measure. If we assume that $||\epsilon||$ is small, then the polarization $\delta_{G,o}$ calculated based on the available opinion measurements o is close to the polarization $\delta_{G,\hat{o}}$ calculated based on the underlying true opinions $\hat{o}$. In the derivation below, we show that

$$|\delta_{G,o} - \delta_{G,\hat{o}}| \leq ||\epsilon|| \mu_{\min}^{-1/2}(L), \tag{1}$$

where $\mu_{\min}(L)$ is the smallest nonzero Laplacian eigenvalue, which is a fixed number for a given network. The error in our polarization measure is thus at most proportional to the measurement error $||\epsilon||$.

The bound (1) follows from the assumption that $\|\epsilon\|$ is small:

$$
\begin{aligned}
\delta_{G,\hat{o}} - \delta_{G,o} &= \sqrt{(\hat{o}^+ - \hat{o}^-)^T L^\dagger (\hat{o}^+ - \hat{o}^-)} - \sqrt{(\hat{o}^+ - \hat{o}^- + \epsilon)^T L^\dagger (\hat{o}^+ - \hat{o}^- + \epsilon)} \\
&= \delta_{G,\hat{o}} \left(1 - \sqrt{1 + \frac{2\epsilon^T L^\dagger (\hat{o}^+ - \hat{o}^-) + \epsilon^T L^\dagger \epsilon}{\delta^2_{G,\hat{o}}}} \right) \\
&= \delta_{G,\hat{o}} \left(1 - \sqrt{1 + \frac{2\epsilon^T L^\dagger (\hat{o}^+ - \hat{o}^-)}{\delta^2_{G,\hat{o}}}} \right) \quad \text{(step 1)} \\
&= \delta_{G,\hat{o}} \left(1 - \left[1 + \frac{\epsilon^T L^\dagger (\hat{o}^+ - \hat{o}^-)}{\delta^2_{G,\hat{o}}} \right] \right) \quad \text{(step 2)} \\
\Rightarrow |\delta_{G,\hat{o}} - \delta_{G,o}| &= \left| \frac{\epsilon^T L^\dagger (\hat{o}^+ - \hat{o}^-)}{\delta_{G,\hat{o}}} \right| \\
&\leq \sqrt{\epsilon^T L^\dagger \epsilon} \quad \text{(step 3)} \\
&\leq \|\epsilon\| \mu_{\min}^{-1/2}(L) \quad \text{(step 4).}
\end{aligned}
$$

In step 1, we use the assumption that ϵ is small, such that $\epsilon^T L^\dagger \epsilon$ is negligible with respect to the other terms. In step 2, we use the Taylor expansion $\sqrt{1+x} = 1 + \frac{x}{2} + O(x^2)$ in combination with the small-error assumption. In step 3, we invoke the Cauchy-Schwarz inequality $\epsilon^T L^\dagger (\hat{o}^+ - \hat{o}^-) \leq \sqrt{\epsilon^T L^\dagger \epsilon} \sqrt{(\hat{o}^+ - \hat{o}^-)^T L^\dagger (\hat{o}^+ - \hat{o}^-)}$. Step 4, finally, follows from the fact that the largest eigenvalue of $L^\dagger$ is the inverse of the smallest nonzero eigenvalue of the pseudoinverse Laplacian, as $\mu_{\max}(L^\dagger) = \mu_{\min}^{-1}(L)$.

8 Twitter & Congress Data

8.1 Summary Statistics

Table S3 shows some summary statistics for the networks extracted from the Twitter data. The table shows, among other things, how well one could partition the network by assigning a node to a community depending on the sign of its o value. We estimate this by calculating the modularity of the partition, which compares the number of edges inside each community with

Network	$|V|$	$|E|$	ℓ	Δ	Q
Obama	204	1,377	2.762	0.183	0.027
Gun Control	1,092	22,471	2.813	0.273	0.176
Abortion	2,211	55,328	2.773	0.190	0.343
VP Debate	5,407	116,249	2.967	0.073	0.364
Second Debate	4,697	94,497	3.115	0.084	0.474
Election	3,965	40,443	3.493	0.088	0.329

Table S3: **Summary statistics of the Twitter debate networks**. For each network, we report: the number of nodes $|V|$, the number of edges $|E|$, the average shortest path length ℓ, the transitivity of the network Δ, and the modularity values Q we would get by bisecting the networks in two communities identified by having a o value of a given sign.

the one we would get with a random network with the same degree distribution as the original one but no communities (*83*). This is another rough estimate of structural separation.

Table S4 shows the same summary statistics for all the US House of Representatives networks. Consistently with what we discuss in the main paper, we can see a noticeable increase in modularity starting at the 99th Congress. We can also see that the share of congressmen with more extreme opinion scores steadily increases over time.

8.2 Polarization Components Correlation

Since our definition of polarization is made of two components – opinion and structure – and their mesoscale interplay, we are implicitly assuming that there is a correlation between them. Here, we test whether this assumption holds on the real-world data gathered on Twitter and the US House of Representatives.

We estimate the opinion component by calculating the average opinion of each side and then averaging their absolute values. For instance, in the 116th Congress, the average positive DW-NOMINATE score was 0.5, while the average negative score was -0.37. We thus infer that $\mu_{116} = (0.5 + 0.37)/2 = 0.43$.

The structural component p_{out} comes from counting the number of edges across communi-

Congress	$\|V\|$	$\|E\|$	ℓ	Δ	Q	$\|o\| > .5$
81	423	39,735	1.592	0.761	0.309	0.070
82	425	43,446	1.549	0.782	0.315	0.066
83	419	36,988	1.625	0.782	0.308	0.067
84	421	40,632	1.576	0.749	0.311	0.076
85	415	38,378	1.590	0.733	0.295	0.082
86	426	42,151	1.563	0.798	0.283	0.095
87	418	39,344	1.598	0.785	0.325	0.083
88	417	39,870	1.590	0.810	0.359	0.086
89	417	39,923	1.588	0.820	0.275	0.091
90	419	36,292	1.638	0.751	0.285	0.081
91	398	33,925	1.613	0.717	0.169	0.087
92	407	35,908	1.599	0.738	0.223	0.091
93	413	40,908	1.559	0.757	0.214	0.104
94	411	41,328	1.549	0.791	0.219	0.105
95	413	41,547	1.546	0.771	0.200	0.108
96	419	43,847	1.540	0.782	0.260	0.116
97	411	39,776	1.571	0.758	0.288	0.119
98	427	49,006	1.499	0.843	0.298	0.114
99	425	46,155	1.558	0.880	0.351	0.120
100	421	45,810	1.556	0.899	0.341	0.120
101	425	46,410	1.562	0.876	0.321	0.130
102	431	48,138	1.546	0.907	0.346	0.131
103	430	48,355	1.516	0.950	0.405	0.155
104	427	45,443	1.621	0.956	0.431	0.189
105	426	44,595	1.700	0.958	0.465	0.194
106	427	44,713	1.593	0.930	0.458	0.192
107	427	44,693	1.590	0.947	0.467	0.203
108	429	45,463	1.807	0.987	0.486	0.203
109	429	45,607	1.739	0.985	0.487	0.220
110	422	44,340	1.821	0.989	0.481	0.219
111	423	46,515	1.544	0.953	0.416	0.214
112	432	47,600	1.520	0.960	0.435	0.286
113	424	44,899	1.835	0.990	0.485	0.295
114	433	47,662	1.546	0.992	0.461	0.315
115	432	47,079	1.587	0.981	0.470	0.327
116	427	45,900	1.546	0.988	0.475	0.296

Table S4: **Summary statistics of the US House of Representatives networks**. Same legend as Table S3. The additional $|o| > .5$ columns reports the share of congressmen with an opinion score outside the $[-0.5, 0.5]$ interval.

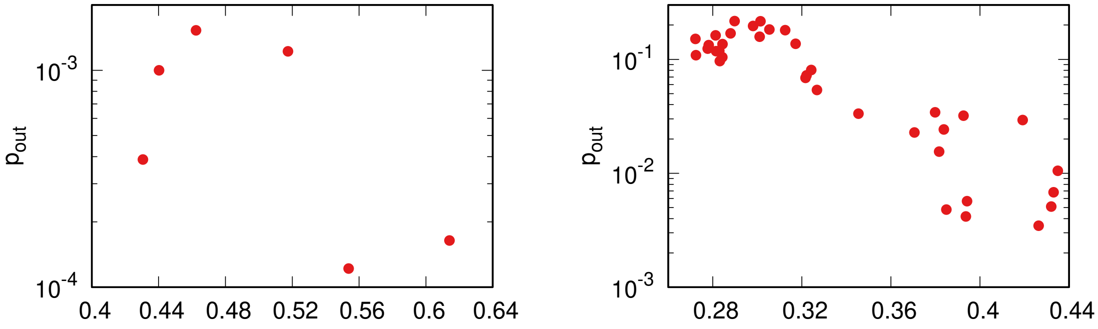

Figure S9: **The correlation between the opinion and structural components of polarization on real-world networks**. Opinion divergence on the x axis, structural separation on the y axis. (Left) Twitter, (Right) US House of Representatives.

ties over the potential number of possible edges across communities, i.e., the number of node pairs belonging to different communities. We employ a Monte Carlo Markov Chain approach for the inference of stochastic block models (*84*) (SBM).

We are unable to estimate the opinion-structural mesoscale interplay, which we indicate with n. This is because real-world data does not organize as neatly as our synthetic experiments. Even if there are multiple communities, they will always have some degree of interconnectedness. It follows that there is no easy way to calculate n, and since finding an appropriate way goes beyond the scope of this paper, we skip the estimation of this parameter.

Figure S9 shows the relationship between μ and p_{out} on all real-world networks we study. We observe the expected negative relationship. High μ correspond to high opinion divergence which we expect to cause a high structural separation by isolating the communities, causing a low value for p_{out}, which is the probability of edges appearing across communities.

The relationship is exponential (note the logarithmic scale in the y axis). In the US House of Representatives network, we obtain a -0.83 Pearson correlation coefficient and a -0.78 Spearman correlation coefficient, both significant as $p < 0.001$. We do not obtain significant coefficients for the Twitter networks, but this is most likely due to the small sample size. The

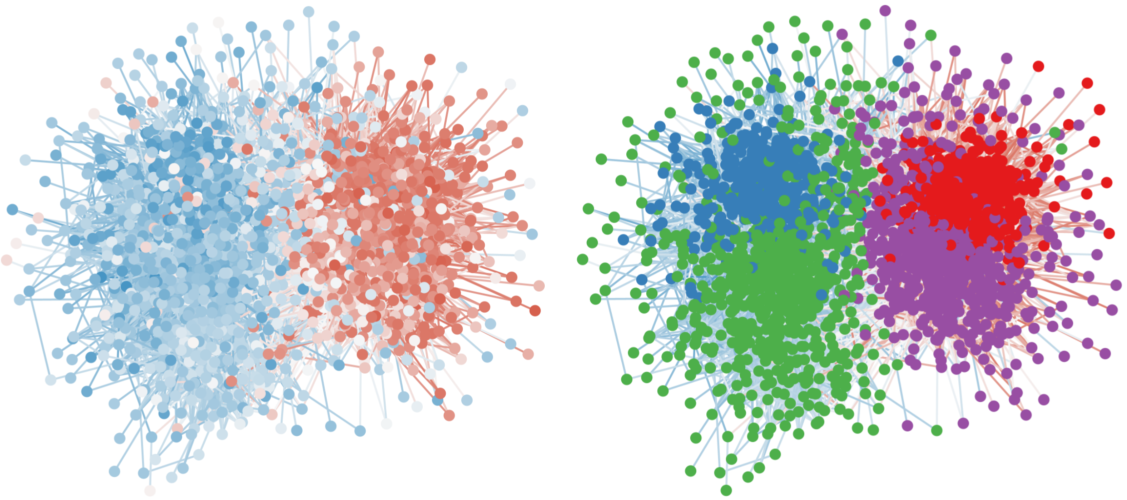

Figure S10: **The communities in the Twitter abortion network**. In both networks, nodes are Twitter users, connected by an edge if the two nodes interacted. The edge color is the average opinion value of the two connected nodes. The two networks have the exact same layout. (Left) Node color is the o value of the node (from -1 dark blue, to $+1$ dark red). (Right) Node color is the inferred community. Edge color is the same as in the left network.

coefficients for the Pearson and Spearman correlations are still negative and equal to -0.45 and -0.6, respectively.

We can conclude that indeed the structural and opinion components of our polarization definition are correlated and reinforce each other.

8.3 Communities in the Twitter Abortion Debate Network

In the main paper we argue that one of the reasons for the Twitter abortion debate network to score higher $\delta_{G,o}$ values is the fact that the network has four nested communities rather than two flat ones. Here, we provide support for this statement. To do so, we infer the community structure of the network using the same method we used in the previous section.

Figure S10 shows the result. On the left, we depict the network as it is in Figure 5 of the main paper. We can see that both the blue and the red community in the network have subcommunities, with brighter and lighter hues, showing a distinction in interaction patterns

	blue	green	purple	red
blue	0.0981	0.0078	0.0009	0.0018
green	0.0078	0.0391	0.0019	0.0003
purple	0.0009	0.0019	0.0614	0.0219
red	0.0018	0.0003	0.0219	0.1393

Table S5: **The edge connection probabilities for the communities in the Twitter abortion network**. Each cell and its color reports the probability that a node in one block connects to a node in another block.

Community	AVG o
blue	-0.52
green	-0.40
purple	0.40
red	0.56

Table S6: **The average opinion value for the Twitter abortion communities**. Each cell – and its color – reports the average o values for the nodes in each block in the Twitter abortion network.

between moderates and extremists. This is confirmed by the SBM community inference, which is shown on the right of Figure S10. Here, we can see that both the left and the right side are further split in two communities which, upon visual inspection, overlap with the brighter and darker hues on either side.

This is not just a visual artifact. Table S5 reports the probability of a block community to connect to another block community. We can see that the diagonal entries for communities paired on the same side of the network are higher than the ones with their community companion. This means that, e.g., the green community is more tightly knit with itself than with the blue community. If the network had only two communities and this hierarchical division was an artifact, there should be no difference in the edge probabilities between the green and blue block. The fact that we see such a difference shows that the network indeed has a mesoscale organization in four communities.

The communities also have distinctive opinion values. Table S6 shows the average o value

for the nodes in each community. Again, we can see noticeable differences, showing that indeed each community is distinct from the others.

9 Supplementary Material Captions

Supplementary File S1. This is a zip file containing all the code necessary to reproduce the results in the paper. Data available at the links provided in the README file. The archive is also available at `https://www.michelecoscia.com/?page_id=2105`.

REFERENCES AND NOTES

1. L. A. Adamic, N. Glance, The political blogosphere and the 2004 U.S. election: Divided they blog, in *Proceedings of the 3rd International Workshop on Link Discovery* (2005), Chicago, Illinois, 21 to 25 August 2005, pp. 36–43.

2. M. Conover, J. Ratkiewicz, M. Francisco, B. Goncalves, F. Menczer, A. Flammini, Political polarization on Twitter, in *Proceedings of the International AAAI Conference on Web and Social Media* (2011), Barcelona, Catalonia, Spain, 17 to 21 July 2011, vol. 5, pp. 89–96.

3. Y. Mejova, A. X. Zhang, N. Diakopoulos, C. Castillo, Controversy and sentiment in online news. arXiv:1409.8152 [cs.CY] (29 September 2014).

4. W. Quattrociocchi, A. Scala, C. R. Sunstein, "Echo chambers on Facebook" (2016); https://dx.doi.org/10.2139/ssrn.2795110.

5. M. Coscia, L. Rossi, How minimizing conflicts could lead to polarization on social media: An agent-based model investigation. *PLOS ONE* **17**, e0263184 (2022).

6. E. Pariser, *The Filter Bubble: What the Internet is hiding from you* (Penguin UK, 2011).

7. H. F. de Arruda, *et al.* , *Inform. Sci.* (2021).

8. F. Cinus, M. Minici, C. Monti, F. Bonchi, The effect of people recommenders on echo chambers and polarization. *Proc. Int. AAAI Conf. Weblogs and Soc. Media* **16**, 90–101 (2022).

9. M. Gentzkow, *Toulouse Network for Information Technology Whitepaper* (2016), pp. 1–23.

10. L. Boxell, M. Gentzkow, J. M. Shapiro, Greater Internet use is not associated with faster growth in political polarization among US demographic groups. *Proc. Natl. Acad. Sci. U.S.A.* **114**, 10612–10617 (2017).

11. E. Kubin, C. von Sikorski, The role of (social) media in political polarization: A systematic review. *Ann. Int. Commun. Assoc.* **45**, 188–206 (2021).

12. S. Iyengar, Y. Lelkes, M. Levendusky, N. Malhotra, S. J. Westwood, The origins and consequences of affective polarization in the United States. *Annu. Rev. Polit. Sci.* **22**, 129–146 (2019).

13. Y. Lelkes, Mass polarization: Manifestations and measurements. *Public Opin. Q.* **80**, 392–410 (2016).

14. M. P. Fiorina, S. J. Abrams, Political polarization in the American public. *Annu. Rev. Polit. Sci.* **11**, 563–588 (2008).

15. A. I. Abramowitz, K. L. Saunders, Is polarization a myth? *J. Polit.* **70**, 542–555 (2008).

16. J. N. Druckman, M. S. Levendusky, What do we measure when we measure affective polarization? *Public Opin. Q.* **83**, 114–122 (2019).

17. N. Dias, Y. Lelkes, The nature of affective polarization: Disentangling policy disagreement from partisan identity. *Am. J. Polit. Sci.* **66**, 775–790 (2022).

18. Y. Lelkes, Policy over party: Comparing the effects of candidate ideology and party on affective polarization. *Polit. Sci. Res. Methods* **9**, 189–196 (2021).

19. L. V. Orr, G. A. Huber, The policy basis of measured partisan animosity in the United States. *Am. J. Polit. Sci.* **64**, 569–586 (2020).

20. F. Bonchi, E. Galimberti, A. Gionis, B. Ordozgoiti, G. Ruffo, Discovering Polarized Communities in Signed Networks, in *Proceedings of the 28th ACM International Conference on Information and Knowledge Management* (2019), Beijing, China, 3 to 7 November 2019, pp. 961–970.

21. R.-C. Tzeng, B. Ordozgoiti, A. Gionis, Discovering conflicting groups in signed networks, in *Advances in Neural Information Processing Systems 33* (NeurIPS 2020), virtual, 6 to 12 December 2020.

22. Z. Huang, A. Silva, A. Singh, in *Proceedings of the Fifteenth ACM International Conference on Web Search and Data Mining* (WSDM'22), Virtual Event, AZ, USA, 21 to 25 February 2022, pp. 390–400.

23. D. Baldassarri, S. E. Page, The emergence and perils of polarization. *Proc. Natl. Acad. Sci. U.S.A.* **118**, e2116863118 (2021).

24. M. Coscia, Generalized Euclidean measure to estimate network distances. *Proc. Innov. Appl. Artif. Intell. Conf.*, **14**, 119–129 (2020).

25. I. Waller, A. Anderson, Quantifying social organization and political polarization in online platforms. *Nature* **600**, 264–268 (2021).

26. A. J. Morales, J. Borondo, J. C. Losada, R. M. Benito, Measuring political polarization: Twitter shows the two sides of Venezuela. *Chaos* **25**, 033114 (2015).

27. C. Musco, I. Ramesh, J. Ugander, R. T. Witter, How to quantify polarization in models of opinion dynamics. arXiv:2110.11981 [cs.SI] (26 October 2021).

28. M. Coletto, K. Garimella, A. Gionis, C. Lucchese, Automatic controversy detection in social media: A content-independent motif-based approach. *Online Soc. Netw. Media* **3–4**, 22–31 (2017).

29. M. Coletto, K. Garimella, A. Gionis, C. Lucchese, A motif-based approach for identifying controversy. *Proc. Int. AAAI Conf. Weblogs Soc. Media* **11**, 496–499 (2017).

30. M. E. Newman, Mixing patterns in networks. *Phys. Rev. E* **67**, 026126 (2003).

31. M. Coscia, The atlas for the aspiring network scientist. arXiv:2101.00863 [cs.CY] (8 February 2021).

32. B. Mønsted, S. Lehmann, Characterizing polarization in online vaccine discourse—A large-scale study. *PLOS ONE* **17**, e0263746 (2022).

33. W. Cota, S. C. Ferreira, R. Pastor-Satorras, M. Starnini, Quantifying echo chamber effects in information spreading over political communication networks. *EPJ Data Sci.* **8**, 35 (2019).

34. M. Cinelli, G. D. F. Morales, A. Galeazzi, W. Quattrociocchi, M. Starnini, The echo chamber effect on social media. *Proc. Natl. Acad. Sci. U.S.A.* **118** e2023301118 (2021).

35. L. Peel, J.-C. Delvenne, R. Lambiotte, Multiscale mixing patterns in networks. *Proc. Natl. Acad. Sci. U.S.A.* **115**, 4057–4062 (2018).

36. P. Guerra, W. Meira Jr., C. Cardie, R. Kleinberg, A measure of polarization on social media networks based on community boundaries. *Proc. Int. AAAI Conf. Weblogs Soc Media* **7**, 215–224 (2013).

37. K. Garimella, G. D. F. Morales, A. Gionis, M. Mathioudakis, Quantifying controversy on social media. *ACM Trans. Soc. Comput.* **1**, 1–27 (2018).

38. K. Garimella, *et al.* , Polarization on social media (2018).

39. K. Darwish, in *International Conference on Social Informatics* (Springer, 2019), pp. 188–201.

40. H. Emamgholizadeh, M. Nourizade, M. S. Tajbakhsh, M. Hashminezhad, F. N. Esfahani, A framework for quantifying controversy of social network debates using attributed networks: Biased random walk (BRW). *Soc. Netw. Anal. Min.* **10**, 90 (2020).

41. A. Cossard, G. de Francisci Morales, K. Kalimeri, Y. Mejova, D. Paolotti, M. Starnini, Falling into the echo chamber: The Italian vaccination debate on Twitter. *Proc. Int. AAAI Conf. Weblogs Soc. Media* **14**, 130–140 (2020).

42. S. Haddadan, C. Menghini, M. Riondato, E. Upfal, RePBubLik: Reducing Polarized Bubble Radius with Link Insertions, in *Proceedings of the 14th ACM International Conference on Web Search and Data Mining* (WSDM'21), Virtual Event, Israel, 8 to 12 March 2021, pp. 139–147.

43. N. E. Friedkin, E. C. Johnsen, Social influence and opinions. *J. Mat. Soc.* **15**, 193–206 (1990).

44. A. Matakos, E. Terzi, P. Tsaparas, Measuring and moderating opinion polarization in social networks. *Data Min. Knowl. Discov.* **31**, 1480–1505 (2017).

45. C. Musco, C. Musco, C. E. Tsourakakis, Minimizing Polarization and Disagreement in Social Networks, in *Proceedings of the 2018 World Wide Web Conference* (WWW'18), Lyon, France, 23 to 27 April 2018, pp. 369–378.

46. D. Chong, J. N. Druckman, Dynamic public opinion: Communication effects over time. *Am. Polit. Sci. Rev.* **104**, 663–680 (2010).

47. J. N. Druckman, T. J. Leeper, Is public opinion stable? Resolving the micro/macro disconnect in studies of public opinion. *Daedalus* **141**, 50–68 (2012).

48. J. Zaller, *The Nature and Origins of Mass Opinion* (Cambridge Univ. Press, 2012).

49. X. Chen, J. Lijffijt, T. De Bie, Quantifying and minimizing risk of conflict in social networks, in *Proceedings of the 24th ACM SIGKDD International Conference on Knowledge Discovery & Data Mining* (KDD'18), London, United Kingdom, 19 to 23 August 2018, pp. 1197–1205.

50. S. Benslimane, J. Azé, S. Bringay, M. Servajean, C. Mollevi, in *International Conference on Web Information Systems Engineering* (Springer, 2021), pp. 339–354.

51. K. Garimella, G. De Francisci Morales, A. Gionis, M. Mathioudakis, Political discourse on social media: Echo chambers, gatekeepers, and the price of bipartisanship, in *Proceedings of the 2018 World Wide Web Conference* (2018), Lyon, France, 23 to 27 April 2018, pp. 913–922.

52. K. Devriendt, S. Martin-Gutierrez, R. Lambiotte, Variance and covariance of distributions on graphs. *SIAM Rev.* **64**, 343–359 (2022).

53. F. Baumann, P. Lorenz-Spreen, I. M. Sokolov, M. Starnini, Modeling echo chambers and polarization dynamics in social networks. *Phys. Rev. Lett.* **124**, 048301 (2020).

54. C. B. Currin, S. V. Vera, A. Khaledi-Nasab, Depolarization of echo chambers by random dynamical nudge. *Sci. Rep.* **12**, 9234 (2022).

55. M. W. Macy, M. Ma, D. R. Tabin, J. Gao, B. K. Szymanski, Polarization and tipping points. *Proc. Natl. Acad. Sci. U.S.A.* **118**, e2102144118 (2021).

56. F. P. Santos, Y. Lelkes, S. A. Levin, Link recommendation algorithms and dynamics of polarization in online social networks. *Proc. Natl. Acad. Sci. U.S.A.* **118**, e2102141118 (2021).

57. M. Coscia, A. Gomez-Lievano, J. Mcnerney, F. Neffke, The node vector distance problem in complex networks. *ACM Comput. Surv.* **53**, 1–27 (2020).

58. D. J. Klein, M. Randić, Resistance distance. *J. Math. Chem.* **12**, 81–95 (1993).

59. L. Akoglu, Quantifying political polarity based on bipartite opinion networks. *Proc. Int. AAAI Conf. Weblogs Soc. Media* **8**, 2–11 (2014).

60. J. B. Lewis, K. Poole, H. Rosenthal, A. Boche, A. Rudkin, L. Sonnet (2019); https://voteview.com/ [accessed 25 February 2022].

61. K. T. Poole, H. Rosenthal, *Congress: A Political-Economic History of Roll Call Voting* (Oxford Univ. Press on Demand, 2000).

62. Z. P. Neal, A sign of the times? Weak and strong polarization in the U.S. Congress, 1973–2016. *Soc. Netw.* **60**, 103–112 (2020).

63. M. Coscia, Pearson correlations on complex networks. *J. Complex Netw.* **9**, cnab036 (2021).

64. M. E. Del Valle, R. B. Bravo, Echo chambers in parliamentary Twitter networks: The Catalan case. *Int. J. Commun.* **12**, 21 (2018).

65. M. Esteve Del Valle, M. Broersma, A. Ponsioen, Political interaction beyond party lines: Communication ties and party polarization in parliamentary Twitter networks. *Soc. Sci. Comput. Rev.* **40**, 736–755 (2022).

66. D. Baldassarri, A. Gelman, Partisans without constraint: Political polarization and trends in american public opinion. *Am. J. Sociol.* **114**, 408–446 (2008).

67. A. Abramowitz, K. Saunders, *The Forum* (De Gruyter, 2005), vol. 3, pp. 1–22.

68. A. Abramowitz, *The Disappearing Center: Engaged Citizens, Polarization, and American Democracy* (Yale Univ. Press, 2010).

69. C. Hare, K. T. Poole, The polarization of contemporary American politics. *Polity* **46**, 411–429 (2014).

70. M. Coscia, *ACM Transactions on Knowledge Discovery from Data (TKDD)* (2022).

71. N. K. Vishnoi, Lx = b. *Found. Trends Theor. Comput. Sci.* **8**, 1–141 (2013).

72. K. Deweese, *Bridging the Theory-Practice Gap of Laplacian Linear Solvers* (University of California, Santa Barbara, 2018).

73. R. I. Kondor, J. Lafferty, Diffusion Kernels on Graphs and Other Discrete Structures, in *Proceedings of the 19th International Conference on Machine Learning* (ICML'2002), Sydney Australia, 8 to 12 July 2002, vol. 2002, pp. 315–322.

74. P. W. Holland, K. B. Laskey, S. Leinhardt, Stochastic blockmodels: First steps. *Soc. Netw.* **5**, 109–137 (1983).

75. P. Erdős, A. Rényi, On the evolution of random graphs. *Publ. Math. Inst. Hung. Acad. Sci* **5**, 17–61 (1960).

76. H. Lu, J. Caverlee, W. Niu, BiasWatch: A lightweight system for discovering and tracking topic-sensitive opinion bias in social media, in *Proceedings of the 24th ACM International on Conference on Information and Knowledge Management* (CIKM'2015), pp. 213–222.

77. L. Wrubel, D. Kerchner, 2020 United States Presidential Election (2021).

78. C. Andris, D. Lee, M. J. Hamilton, M. Martino, C. E. Gunning, J. A. Selden, The Rise of partisanship and super-cooperators in the U.S. House of Representatives. *PLOS ONE* **10**, e0123507 (2015).

79. G. Karypis, V. Kumar, METIS: A software package for partitioning unstructured graphs, partitioning meshes, and computing fill-reducing orderings of sparse matrices (1997).

80. M. Coscia, in *Proceedings of the 2019 IEEE/ACM International Conference on Advances in Social Networks Analysis and Mining* (ASONAM'2019), Vancouver, British Columbia, Canada, 27 to 30 August 2019, pp. 1–8.

81. R. Pastor-Satorras, C. Castellano, P. Van Mieghem, A. Vespignani, Epidemic processes in complex networks. *Rev. Mod. Phys.* **87**, 925–979 (2015).

82. S. T. McCormick, M. R. Rao, G. Rinaldi, Easy and difficult objective functions for max cut. *Math. Prog.* **94**, 459–466 (2003).

83. M. E. Newman, Modularity and community structure in networks. *Proc. Natl. Acad. Sci. U.S.A.* **103**, 8577–8582 (2006).

84. T. P. Peixoto, Efficient Monte Carlo and greedy heuristic for the inference of stochastic block models. *Phys. Rev. E* **89**, 012804 (2014).
